# Psychometric Properties of the German Version of the Quality of Life after Brain Injury Scale for Kids and Adolescents (QOLIBRI-KID/ADO) Using Item Response Theory Framework: Results from the Pilot Study

**DOI:** 10.3390/jcm12113716

**Published:** 2023-05-27

**Authors:** Marina Zeldovich, Katrin Cunitz, Sven Greving, Holger Muehlan, Fabian Bockhop, Ugne Krenz, Dagmar Timmermann, Inga K. Koerte, Philine Rojczyk, Maike Roediger, Michael Lendt, Nicole von Steinbuechel

**Affiliations:** 1 Institute of Medical Psychology and Medical Sociology, University Medical Center Göttingen, Waldweg 37A, 37073 Goettingen, Germany; 2 Department Health & Prevention, Institute of Psychology, University of Greifswald, Robert-Blum-Str. 13, 17489 Greifswald, Germany; 3 Department of Child and Adolescent Psychiatry, Psychosomatics, and Psychotherapy, Ludwig-Maximilians-Universität München, 80336 Munich, Germany; 4 Department of Pediatric and Adolescent Medicine-General Pediatrics-Intensive Care Medicine and Neonatology, University Hospital Muenster, Albert-Schweitzer-Campus 1, 48149 Muenster, Germany; 5 Neuropediatrics, St. Mauritius Therapeutic Clinic, Strümper Straße 111, 40670 Meerbusch, Germany

**Keywords:** pediatric traumatic brain injury (TBI), health-related quality of life (HRQOL), patient-reported outcome measure (PROM), item response theory (IRT)

## Abstract

Health-related quality of life (HRQOL) is an important indicator for recovery after pediatric TBI. To date, there are a few questionnaires available for assessing generic HRQOL in children and adolescents, but there are not yet any TBI-specific measures of HRQOL that are applicable to pediatric populations. The aim of the present study was to examine psychometric characteristics of the newly developed Quality of Life After Brain Injury Scale for Kids and Adolescents (QOLIBRI-KID/ADO) questionnaire capturing TBI-specific HRQOL in children and adolescents using an item response theory (IRT) framework. Children (8–12 years; *n* = 152) and adolescents (13–17 years; *n* = 148) participated in the study. The final version of the QOLIBRI-KID/ADO, comprising 35 items forming 6 scales, was investigated using the partial credit model (PCM). A scale-wise examination for unidimensionality, monotonicity, item infit and outfit, person homogeneity, and local independency was conducted. The questionnaire widely fulfilled the predefined assumptions, with a few restrictions. The newly developed QOLIBRI-KID/ADO instrument shows at least satisfactory psychometric properties according to the results of both classical test theoretical and IRT analyses. Further evidence of its applicability should be explored in the ongoing validation study by performing multidimensional IRT analyses.

## 1. Introduction

Pediatric traumatic brain injury (TBI) is a leading cause of death and disability among children and adolescents worldwide [1]. It represents a substantial burden for those affected and their next of kin. Short- or long-time consequences cover a broad range of functional [2], cognitive [3], developmental [2], and behavioral [4] problems and impair health-related quality of life (HRQOL) [5]. In particular, moderate to severe TBI in children and adolescents can hamper family functioning longitudinally [6]. 

HRQOL is an important indicator for recovery after adult and pediatric TBI [7]. One can distinguish between generic and disease-specific HRQOL instruments. Generic instruments typically cover a broader area of health [7], whereas specific instruments focus on a particular disease state [8]. The advantage of disease-specific instruments over generic ones is that they are able to capture the consequences of a specific health condition [8] and are therefore more sensitive [9,10]. To date, a number of patient-reported measures (PROMs) exist for assessing generic HRQOL in children and adolescents (e.g., Pediatric Quality of Life Inventory, PedsQL [11]; KIDSSCREEN-52 [12] and its short versions). However, there is not yet any PROM designed to measure TBI-specific pediatric HRQOL. Hence, the provision of TBI-specific instruments for children and adolescents would facilitate the understanding of the consequences of TBI in the pediatric population. 

To assess TBI-specific HRQOL in adults, the Quality of Life after Brain Injury (QOLIBRI) self-report instrument comprising 37 items has been developed [13,14]. This instrument has been translated into more than 23 languages [13,15,16,17] and finds its application across the entire spectrum of TBI severity. The QOLIBRI was used as a theoretical starting point for developing a questionnaire measuring TBI-specific HRQOL in pediatric TBI populations: the QOLIBRI for kids and adolescents (QOLIBRI-KID/ADO). The aim of the study project was to develop, psychometrically test, and validate an instrument tailored for children (8–12 years) and adolescents (13–17 years) in order to be able to capture TBI-specific HRQOL from childhood to an advanced age. 

The selection of items for the final item set of the QOLIBRI-KID/ADO involved two levels: the content and the psychometric level. Concerning both, we tried to achieve comparability to the adults’ QOLIBRI version yet adapted for application in children and adolescents. We chose items from the QOLIBRI and an item pool consisting of over 300 items collected from questionnaires measuring HRQOL and related constructs. The final questionnaire comprising 35 items was investigated using the classical test theory (CTT; e.g., McDonald, 1999 [18]) framework. The questionnaire showed satisfactory results for the item and scale level, with Cronbach’s α ranging from 0.70 to 0.89 [19]. The interclass correlation coefficients for the scales were widely acceptable (0.42 to 0.64) and the six-factorial structure comprising six scales could be retained. For more details on questionnaire development and its psychometric characteristics, see von Steinbuechel et al. [19]. 

Item response theory (IRT) as a complementary approach to the CTT framework has several advantages [20] in the domain of patient-reported outcomes (PRO) [21]. As part of IRT, Rasch models allow for a simultaneous estimation of item and person parameters, a visualization of response patterns and item difficulties considering the distribution of person traits with item or category characteristic curves (ICC/CCC), and accounting for different response behavior across groups of interest by means of differential item functioning (DIF). The IRT-based approaches also allow for a more precise description of the item and scale fit of a PROM [21]. 

Given the mentioned advantages of the IRT, the aim of the present study was to analyze the data obtained from the pilot study on the development of the QOLIBRI-KID/ADO using IRT approaches besides classical test theoretical analyses, which have recently been carried out [19]. 

## 2. Materials and Methods

### 2.1. Participants

Data collection took place between January 2019 and January 2022. Of the approximately 5000 families contacted by postal mail using records from 11 medical centers in Germany, 300 responded and met the inclusion criteria for participating in the study. Inclusion criteria were the following: age between 8 and 17 years, diagnosis of TBI (at least 3 months but not more than 10 years before study enrollment), information on the TBI severity (obtained from the Glasgow Coma Scale [GCS] [22] or medical reports), and the ability to understand and fill in a questionnaire. Children and adolescents suffering from epilepsy, severe polytrauma, serious mental illness prior to the TBI, or disease leading to death were excluded from this study. Written informed consent was collected from children and/or their parents at the time of study enrollment. 

**Figure 1:**
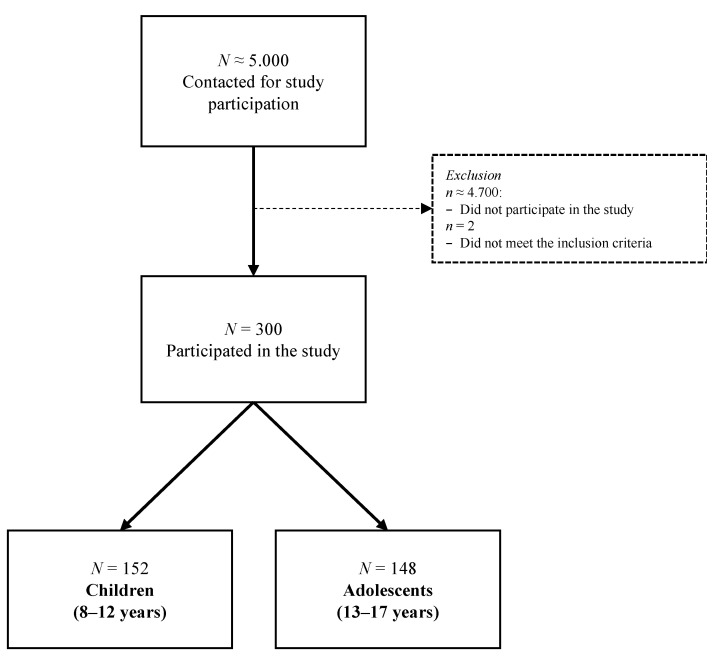
Sample attrition.

Overall, 152 children (8–12 years) and 148 adolescents (13–17) were included in the final study sample. Most participants were interviewed in a face-to-face offline setting (8–12 years: *n* = 113; 13–17 years: *n* = 111) and the remaining were tested face-to-face online (8–12 years: *n* = 39; 13–17 years: *n* = 37). Parents filled in respective proxy paper–pencil forms. For an overview on sample attrition, see Figure 1.

### 2.2. Ethical Approval

The QOLIBRI-Kid/Ado study was conducted in accordance with all relevant laws of Germany including but not limited to the ICH Harmonized Tripartite Guideline for Good Clinical Practice (“ICH GCP”) and the World Medical Association Declaration of Helsinki (“Ethical Principles for Medical Research Involving Human Subjects”). The study attained ethical clearance at each recruitment center and informed consent for all participants according to the German law for data protection (General Data Protection Regulation, DSGVO). The Ethics Committee of the University Medical Center Göttingen approved the study (application no.: 19/4/18).

### 2.3. Sociodemographic and Injury-Related Data

Participants’ characteristics were gathered at the time of study enrollment. Sociodemographic data comprised information on gender and age. Injury-related information included TBI severity (mild, moderate, severe), number of lesions (no lesions vs. at least one), and time since injury in years. With the Kings Outcome Scale for Childhood Head Injury (KOSCHI) [23], functional recovery after TBI was assessed using the following categories: 1 = ‘dead’, 2 = ‘vegetative state’, 3a = ‘lower severe disability’, 3b = ‘upper severe disability’, 4a = ‘lower moderate disability’, 4b = ‘upper moderate disability’, 5a = ‘good recovery’, or 5b = ‘intact recovery’.

### 2.4. QOLIBRI-Kid/Ado

The newly developed QOLIBRI-KIDO/ADO questionnaire [19] comprises 35 items associated with 6 scales assessing ‘Cognition’ (7 items), Self’ (5 items), ‘Autonomy and Daily Life’ (7 items), ‘Social’ (6 items), ‘Emotions’ (4 items), and ‘Physical’ (6 items) domains. Items are answered on a five-point Likert scale format (‘not at all’, ‘slightly’, ‘moderately’, ‘quite’, ‘very’). Whereas the first four scales assess the satisfaction in the respective domains (i.e., ‘How satisfied are you with…’), the last two scales focus on feelings of being bothered (i.e., ‘How bothered are you with…’) in the respective arears. The calculation of the scale and the total scores comprises a transformation to a 0–100 scale for better interpretation, with higher values indicating better HRQOL. For more details on the scales and items of the questionnaire, see Appendix A, Table A1—QOLIBRI-KID/ADO: Scales and Items. 

### 2.5. Statistical Analyses

First, descriptive statistics for sociodemographic, injury-related, and scale characteristics for both age groups (i.e., children and adolescents) and the total sample were obtained. Then, response patterns based on age groups (i.e., children and adolescents), TBI severity groups (i.e., mild, moderate, and severe TBI), and study setting (i.e., offline vs. online) were investigated. The distribution of the response behavior indicated whether all five response categories were equally chosen by the participants of our study sample.

Considering the ordered ordinal nature of item responses, we applied a unidimensional partial credit model (PCM) [24] to investigate psychometric properties of the QOLIBRI-KID/ADO on the scale level. The PCM is an extension of a dichotomous Rasch model comprising estimation of thresholds, which are also known as item step difficulty parameters [25]. The item parameter (β) measuring item difficulties was estimated using conditional maximum likelihood [25]. Person parameter (θ) measuring latent trait (i.e., the extent of TBI-related HRQOL) estimation occurred using joint maximum likelihood [25]. 

Category characteristic curves (CCC) and person–item maps were used to visualize probabilities of response categories’ endorsements in relation to person parameters and, subsequently, to detect potential threshold-ordering problems. Person–item maps provided summarized information of the item parameters on the scale level. Model assumptions comprising unidimensionality (also referred to as item homogeneity), person homogeneity, local independence, and monotonicity were investigated and evaluated according to the criteria listed below.

*Unidimensionality* assumption postulates that all items of one scale measure the same latent trait as reflected by the person parameter (θ). Unidimensionality was assessed using Martin-Loef’s likelihood ratio test (LRT) [26] applying internal split criterion (i.e., the median). Non-significant test values indicate no violation of unidimensionality assumption.

*Monotonicity* assumption states that, with a higher level of the latent trait (θ), the probability of choosing a higher response category increases monotonically [27,28]. Contrary to previous assumptions [27], the monotonicity assumption also applies to polytomous response formats [28]. Therefore, this was investigated by relating the probability of answering to the latent trait. For this purpose, the so-called restscore was used. The restscore of an item *i* implies the differences between the total score and the score of the item *i*, which is used as an approximation for the person score on the latent trait [29]. The monotonicity assumption was retained if the value did not exceed 0.03 [29].

The items *infit* and *outfit* statistics [30] were calculated to assess the consistency of the response behavior. The infit statistic is sensitive with respect to low variation among participants concerning item endorsement. In contrast, the outfit statistic is sensitive with respect to outlying observations; for example, when participants with a high latent trait endorse frequently low response categories. Evaluation of the results applied the following cut-off values: the expected value was 1 (no deviation between observed and estimated patterns of the response matrix), though values < 0.5 were acceptable considering possible low separation ability of the items; values ranging from 0.5 to 1.5 were satisfactory; values from 1.5 to 2.0 were acceptable; and values > 2 indicated a problem [31]. 

*Person homogeneity* was assessed using the Andersen’s LRT [32] with three different external split criteria (i.e., age: children vs. adolescents; TBI severity: mild vs. moderate/severe; study setting: offline vs. online). This approach allows for the identification of items displaying differential item functioning (DIF) [25]. Non-significant test values indicated no difference between the examined groups, allowing for simulations evolution of the HRQOL across these groups.

*Local independence* was measured using the $Q_{3}$ statistic, which represents the correlation of the residuals among items, i.e., the variance explained by the person parameters (θ) after outpartialization. Under local independence, we expected the value of $Q_{3}$ to be close to zero. In the present study, the more robust statistic, i.e., adjusted $aQ_{3}$ ($Q_{3_{ij}}-Q_{3_{mean}}$), was reported. A value < 0.20 indicated no violations [33]. In line with other research studies on PROMs, we additionally considered values ranging from 0.20 to 0.30 as acceptable [34,35].

All analyses were carried out using R version 4.0.2 [36] under application of the following packages: ‘table1’ [37] for descriptive statistics, *Extended Rasch Modeling* (‘eRm’) [38] for PCM analyses and *Item Analysis in Rasch Models* (‘iarm’) [39] for the PCM-related item analyses, ‘mokken’ [40,41] for monotonicity analyses, and test analysis modules (‘TAM’) [42] for testing local independence. Significance was set at 5% (α=0.05). 

## 3. Results

### 3.1. Sample Characteristics

The study sample contained 152 children (62% males) and 148 adolescents (57% males). Most participants sustained a mild TBI (72%) with no lesions visible on a CT scan (69%). The majority experienced TBI between 4 and 10 years ago, with 76% having been fully functionally recovered when entering the study. For details, see Table 1.

### 3.2. Response Behavior

Participants were more likely to endorse response categories reflecting higher levels of satisfaction or lower levels of being bothered. Therefore, ratings ranging from ‘moderately’ to ‘not all’ in four scales (Cognition, Self, Autonomy & Daily Life, and Social: <5%) and ratings ‘moderately’ to ‘very’ in the scales Emotions and Physical (5–20% dependent on item) were less frequently chosen. Consequently, some items lacked endorsements in the category ‘not at all’ across both age groups (Cognition: ‘Orientation’, Self: ‘Accomplishment’) and in some age and TBI severity groups. For more details, see Appendix B, Table A2, Table A3, Table A4, Table A5, Table A6 and Table A7.

### 3.3. Threshold Disorder

Overall, 15 out of 35 items exhibited no threshold disorders, indicating that the probability of choosing a higher response category increased with an increasing latent trait. For other items, the order of the thresholds was violated mostly at one point and in some cases at two. The Cognition scale was the most affected (six out of seven items), whereas the Self and the Emotion scales each contained only one item that did not meet the requirements. For details on the number of disordered thresholds per item, see Table 2. For threshold parameters, see Appendix C, Table A8.

**Figure 2:**
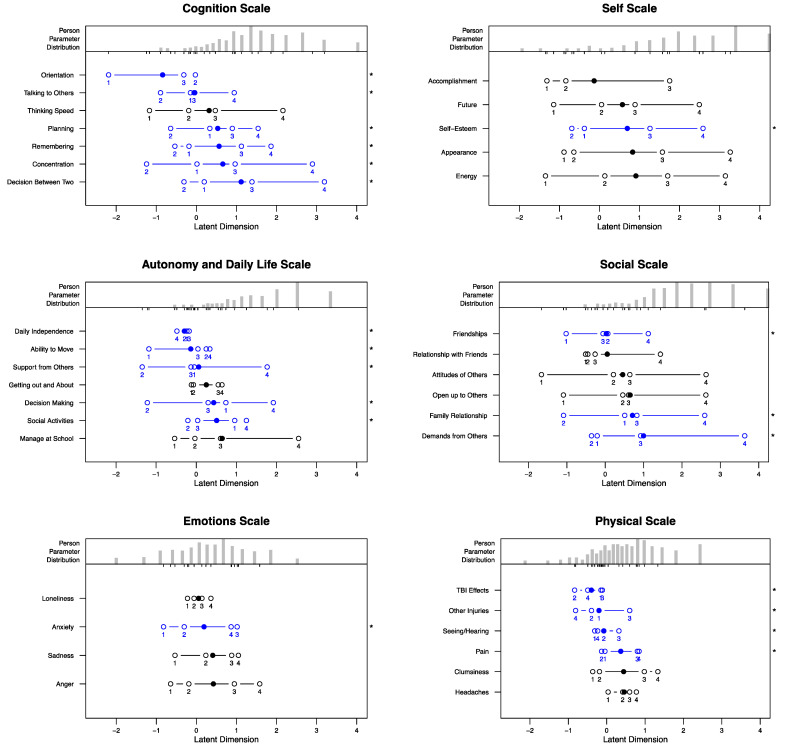
Person–item maps for the QOLIBRI-KID/ADO scales. Upper parts of the respective scales’ graphs depict person parameter (θ) distribution. Lower parts present item thresholds (unfilled circles) and item location parameters or average item parameters (filled circles). Blue-colored items additionally marked with an asterisk (*) indicate thresholds disorder. The order of the items corresponds to their position on the latent trait dimension (from lowest to highest).

Person–item maps provided in Figure 2 visualize threshold parameters in relation to the latent trait for QOLIBRI-KID/ADO scales. The distribution of the person parameters (upper part of respective scale figure) indicates a slight rightward trend toward reporting higher HRQOL in the scales measuring satisfaction. The estimated person parameters of both bothered scales were largely normally distributed. For CCCs of the items, please see Online Supplement S1—Category Characteristic Curves.

### 3.4. Unidimensionality

The unidimensionality assumption could be retained for four scales (Cognition, Self, Autonomy & Daily Life, and Social), showing non-significant results (*p* > 0.05) according to the Martin-Loef’s LRT using an internal split criterion. However, the Emotion and Physical scales revealed a significant violation of the unidimensionality assumption (*p* = 0.008 and *p* = 0.027, respectively). For more details, see Table 2.

### 3.5. Monotonicity

In general, all items of the QOLIBRI-KID/ADO fulfilled monotonicity requirements. Two exceptions were observed concerning the item ‘Anger’ (0.08) (Emotion scale) and the item ‘Seeing/Hearing’ (0.05) (Physical scale), which displayed problems in two and one response categories, respectively. For more details, see Table 2.

### 3.6. Item Fit Statistics

Most of the items (32 out of 35) showed no irregularities regarding both item infit and outfit statistics. Exceptions were the items ‘Appearance’ (infit: 0.765, *p* = 0.040; outfit: 0.786, *p* = 0.048) and ‘Energy’ (infit: 1.302, *p* = 0.006; outfit: 1.304, *p* = 0.002) from the Self scale as well as the infit value of the item ‘Family relationship’ (infit: 1.285, *p* = 0.004) from the Social scale. However, none of the values exceeded the cut-off of 1.5. For more details, see Table 2.

### 3.7. Person Homogeneity and DIF

According to the non-significant results of the Andersen’s LRT (*p* > 0.05), all scales showed no evidence of violation of the person homogeneity assumption, indicating that there is no DIF between children and adolescents. However, due to missing responses in some response categories either in both or in one of the investigated age groups, complete scale analyses were only possible for Emotions and Physical scales. Testing for person homogeneity in Self, Autonomy & Daily Life, and Social scales, was carried out only for those items with exhausted responses.

An investigation of TBI severity groups indicated no presence of DIF in all scales except for the Physical scale, which revealed significant differences (*p* < 0.001). In addition, analyses for the Social scale could not be performed because at least one response was missing for each item either in the group after mild TBI or in the group after moderate/severe TBI.

The assumption could be maintained for the study setting investigations, indicating no difference in response behavior between children interviewed offline and those participating in the study via video calls. All scales except for the Social scale showed non-significant LRT Andersen’s results (*p* < 0.05). The latter was affected by missing responses in one or both groups in five out of six items, so DIF could not be tested. For more details, see Table 2.

### 3.8. Local Independence

The Cognition and Emotion scales fully met the requirements of local independence, with adjusted Q3 scores not reaching the 0.20 value. The other scales exceeded the value of 0.20, but not the cut-off value of 0.30. Residual correlations of the following items were responsible for the slightly increased values: ‘Appearance’ and ‘Self-Esteem’ (−0.23) of the Self scale; ‘Getting out and About’ and ‘Social Activities’ (−0.22) of the Autonomy and Daily Life scale; ‘Attitudes of Others’ and ‘Demands from Others’ (0.25) of the Social scale; as well as ‘TBI effects’ and ‘Pain’ (0.21), ‘TBI effects’ and ‘Clumsiness’ (0.21), ‘Clumsiness’ and ‘Seeing/Hearing’ (0.27), and ‘TBI effects’ and ‘Other Injuries’ (0.29) of the Physical scale. For more details, see Table 2.

## 4. Discussion

The present study examined psychometric properties of the newly developed QOLIBRI-KID/ADO instrument assessing disease-specific HRQOL in children and adolescents after TBI, applying IRT methods. In general, the results suggest that the instrument widely fulfils the assumptions of the polytomous Rasch model. The results show that the QOLIBRI-KID/ADO is equally applicable to children and adolescents. Therefore, aggregate analyses of both age groups as well as longitudinal HRQOL measurements from childhood to adolescence are possible. However, different TBI severity groups in pediatric samples should be treated separately since children and adolescents after mild vs. moderate/severe TBI showed a different probability of endorsing the items measuring physical TBI-related HRQOL. Some further results should be discussed in more detail.

Response categories representing higher HRQOL were more often selected by the participants. This is in line with previous findings in children [43,44,45] and adults [46,47] from TBI and general population samples showing that the distribution of the generic and TBI-specific (HR)QOL scores tends to be skewed in favor of a higher quality of life rating. A closer look at the response patterns within the TBI groups reveals the tendency of the more severely injured participants reporting lower HRQOL. This is again consistent with previously reported research indicating that lower HRQOL associates with greater TBI severity [48]. The distribution of the TBI severity groups in our sample was unequal, with the vast majority having suffered a mild TBI. Additionally, most participants experienced a TBI two to ten years prior to study enrolment. These two facts could partly explain the relatively high level of TBI-specific HRQOL, resulting in the underutilization of response categories that capture impairment.

Disruption of the thresholds refers to the fact that the probability of selecting a higher response category is not always associated with an increase in the latent trait (i.e., TBI-specific HRQOL). A closer look at the order of the thresholds reveals that, in most cases, one threshold per item displayed irregularities. Thereby, the probability of endorsing the second-lowest category was higher than choosing the lowest one. This pattern can again be attributable to the non-exhausted selection of responses and high prevalence of the mild TBI cases in the study sample. However, the analyses of monotonicity indicate that the probability of endorsing a higher response category monotonically increased with a higher level of the latent trait across all items on the scale level, with only very few exceptions in the lowest and highest category of the item ‘Anger’ and the second highest category of the item ‘Seeing/Hearing’.

Item fit statistics of three items showed almost no violations. Despite significant *p*-values, they can still be considered as satisfactory according to the proposed cut-off values. The slightly increased infit and outfit values can also be caused by a relatively low number of observations in some response categories [31].

Combining the results discussed above with the findings on the utilization of the response categories, we conclude that the QOLIBRI-KID/ADO scale scores are widely able to capture TBI-specific HRQOL, with higher values indicating a higher expression of the latent trait. However, further investigation of the psychometric properties of the QOLIBRI-KID/ADO is warranted in more severely injured children and adolescents and in more acute stages of the TBI. In addition, further studies should address the topic of the number of responses in greater detail. Comparing different possible response formats (e.g., three response categories vs. five) in pediatric TBI samples should strengthen the applicability of the response scales of the instrument. 

The positively worded scales assessing satisfaction fulfilled the assumption of unidimensionality, whereas the negatively worded items of the Emotional and Physical scales measuring being bothered with symptoms, impairments, and TBI-related problems did not. These results can probably be explained by different subdomains covered by the items of the scales. In addition to the questions directly related to TBI (e.g., ‘TBI effects’), the Physical scale contains items measuring headaches, other pain, and other injuries. Because impairments in these areas may have a cause other than TBI, this could be reflected in the violation of unidimensionality. For example, chronic headaches and migraines in youths [49], other pains [50] or other injuries (e.g., burns [51] or fractures [52,53]) have been shown to negatively affect generic HRQOL, which may also be relevant for TBI-specific HRQOL. The same explanation can be applied to the Emotion scale, which, in addition to emotional aspects that may be related to affective disturbances, asks about perceived disturbances due to anger. Furthermore, the scale length (i.e., four items) might have influenced the results since the median split, which is dividing the items in two equal groups for further analyses, was used as an internal criterion for unidimensionality testing. 

Although no differences in item parameters were found between the two age groups, items that were excluded due to missing responses in some categories could not be considered. The results of analyses using logistic ordinal regression of differential item functioning (LORDIF) reported by Steinbuechel et al. [19] suggested no differences in responses between children and adolescents when using all items of the questionnaire. Therefore, an aggregation of children’s and adolescents’ responses seems appropriate. Differences in responses between participants with mild and moderate-to-severe TBI regarding the Physical scale may be explained by differences in HRQOL scores according to injury severity [48]. Because the assumption of person homogeneity was only partially maintained in the present study, the authors suggest that the QOLIBRI-KID/ADO needs further validation in future studies for both age and TBI severity groups using the IRT framework. The absence of DIF in relation to the study setting indicates that, at least in our sample, the responses of children and adolescents after TBI were not affected by these (mainly forced by the COVID-19 pandemic) circumstances. This finding may be useful in encouraging researchers to conduct studies online when there is no opportunity for on-site testing. However, careful consideration should be given to whether participants are able to participate online and whether the methods (e.g., measurement tools, questionnaires, and surveys, etc.) are appropriate for an online study setting.

Finally, most items of the QOLIBRI-KID/ADO scales were locally independent. However, responses of some items seem to not be fully independent from each other. In particular, items measuring the feeling of being bothered by consequences of TBI may be associated with other problem areas (i.e., suffering from pains, being clumsy, and having problems caused by other injuries). Given that different types of pain can occur after a TBI and impact the HRQOL [54], this finding is not surprising. Therefore, special attention should be paid to these items in the final validation study. Furthermore, analyses of subgroups (e.g., based on TBI severity) or latent classes may facilitate the identification of characteristics to explain these dependencies.

### 4.1. Strengths and Limitations

The present study is the first study investigating psychometric properties using the IRT framework, which has several aforementioned advantages over classical test theoretical investigations. However, the results should be interpreted with caution. The children and adolescents originally completed questionnaires with 83 and 87 items, respectively, which were used in the pilot study phase. Thus, the final reduced 35-item version of the QOLIBRI-Kid/Ado was not administered directly to the study participants. Longer scales may affect response quality; for example, in terms of motivation [55]. Therefore, a final validation with the 35 items extracted for the final version of the QOLIBRI-KID/ADO is currently ongoing. Thus, we consider the analyses of the present study to be preliminary. Further studies applying the QOLIBRI-KID/ADO should pay particular attention to assumptions met with some restrictions, especially the problem of threshold disorders and the unidimensionality of the scales measuring bother. 

In addition, the response rate was rather low (approximately 6% of those contacted responded and fully met the inclusion criteria). Although the distribution of sociodemographic and clinical characteristics of the TBI population is largely adequately reflected (i.e., predominantly mild TBI cases, more males than females, high recovery rates), the study may have suffered from self-selection bias. Therefore, further validation of this newly developed instrument with samples that more accurately reflect the pediatric TBI population is highly recommended, especially for more severe TBI. This also applies to the patient groups that were excluded due to the study exclusion criteria (i.e., patients with severe systemic diseases, epilepsy, or severe polytrauma). Further research is strongly encouraged to better understand the impact of TBI on HRQOL in these groups.

### 4.2. Future Perspectives

In the final validation study of the QOLIBRI-KID/ADO, the structure of the questionnaire could be investigated using multidimensional IRT approaches, such as the multidimensional random coefficients multinomial logit model [56] or multidimensional mixed Rasch models (e.g., Fischer and Molenaar, 1995 [57]). Such multidimensional models would allow the interdependencies between the scales and the DIF to be examined, while considering the latent structure of the QOLIBRI-KID/ADO. This would allow us to account for the different HRQOL facets being measured by the instrument. Finally, psychometric evidence for the proxy version of the QOLIBRI-KID/ADO using both the CTT and IRT frameworks would provide an alternative way to examine HRQOL after pediatric TBI, especially when children are unable to respond on their own.

## 5. Conclusions

The newly developed QOLIBRI-KID/ADO instrument displays satisfactory psychometric properties according to the results of both the item response and classical test theoretical analyses. Therefore, the application of the instrument in the current form is justified. Further evidence of its applicability should be explored in the final validation study while considering implications that arose from the pilot investigations. Meanwhile, the QOLIBRI-KID/ADO may already find its application in clinical practice and research to assess HRQOL in children and adolescents.

## Figures and Tables

**Table 1:** Sample characteristics.

		Children	Adolescents	Total
		*n* = 152	*n* = 148	*N* = 300
Study setting	Offline	113 (74%)	111 (75%)	224 (75%)
	Online	39 (26%)	37 (25%)	76 (25%)
Gender	Female	58 (38%)	62 (42%)	120 (40%)
	Male	94 (62%)	85 (57%)	179 (60%)
	Diverse	0 (0%)	1 (1%)	1 (%)
TBI severity	Mild	106 (70%)	109 (74%)	215 (72%)
	Moderate	16 (11%)	9 (6%)	25 (8%)
	Severe	30 (20%)	30 (20%)	60 (20%)
Presence of lesion(s)	No	108 (71%)	100 (68%)	208 (69%)
	Yes	43 (28%)	43 (29%)	86 (29%)
	Missing	1 (1%)	5 (3 %)	6 (2%)
KOSCHI disability score	3a	0 (0%)	0 (0%)	0 (%)
	3b	1 (1%)	0 (0%)	1 (%)
	4a	3 (2%)	5 (3%)	8 (3%)
	4b	4 (3%)	18 (12%)	22 (7%)
	5a	15 (10%)	25 (17%)	40 (13%)
	5b	129 (85%)	100 (68%)	229 (76%)
Years since TBI	<1	4 (3%)	3 (2%)	7 (2%)
	1–<2	24 (16%)	20 (14%)	44 (15%)
	2–<4	45 (30%)	36 (24%)	81 (27%)
	4–10	79 (52%)	88 (59%)	167 (56%)
	Missing	0 (0%)	1 (1%)	1 (%)

Note. *n*/*N*: absolute frequency (per group/total), %: relative frequencies, Study setting: face-to-face interviews conducted either offline or online (via video call), KOSCHI: Kings Outcome Scale for Childhood Head Injury, 3b = ‘upper severe disability’, 4a = ‘lower moderate disability’, 4b = ‘upper moderate disability’, 5a = ‘good recovery’, or 5b = ‘intact recovery’, TBI: traumatic brain injury.

**Table 2:** Overview on analyses results.

Scale	Item	# of Response Categories Used	# of Disordered Thresholds	Unidimensionality (Median Split)	Monotonicity	Outfit	Infit	Person Homogeneity						Local Independence (Adjusted Q3)
								(DIF: Age)		(DIF: TBI Severity)		(DIF: Offline vs. Online)		
Desired Results →		5 All Item Responses Exhausted	0 No Disorder (1 < 2 < 3 < 4)	*p* > 0.05 Martin-Loef LRT	<0.03 No Violations	*p*_adj_ > 0.05	*p*_adj_ > 0.05	1 Item Included in the Analyses	*p* > 0.05 AndersenLRT	1 Item Included in the Analyses	*p* > 0.05 AndersenLRT	1 Item Included in the Analyses	*p* > 0.05 AndersenLRT	Adjusted Q3 < 0.20
Cognition (7 items)	Concentration	**5**	1	**LR = 81.75** **df = 179** ***p* > 0.99**	**0**	**0.641**	**0.53**	0 ^d^	*LR = 22.91* *df = 15* *p = 0.086*	**1**	**LR = 24.49** **df = 19** ***p* = 0.178**	**1**	**LR = 31.74** **df = 23** ***p* = 0.106**	**M = 0.00** **(SD = 0.08)** **R: −0.17–0.16**
	Talking to Others	**5**	1		**0**	**>0.99**	**>0.99**	0 ^d^		**1**		**1**		
	Remembering	**5**	1		**0**	**>0.99**	**>0.99**	**1**		**1**		**1**		
	Planning	**5**	1		**0**	**>0.99**	**>0.99**	**1**		**1**		**1**		
	Decision Between Two Things	**5**	1		**0**	**0.641**	**0.53**	**1**		**1**		**1**		
	Orientation	4 ^a^	1		**0**	**>0.99**	**>0.99**	0 ^d^		**1**		**1**		
	Thinking Speed	**5**	**0**		**0**	**0.667**	**0.53**	**1**		**1**		**1**		
Self (5 items)	Energy	**5**	**0**	**LR = 56.96** **df = 87** ***p* = 0.995**	**0**	*0.002 ^c^*	*0.0063*	0 ^d^	*LR = 5.86* *df = 7* *p = 0.557*	**1**	**LR = 26.63** **df = 18** ***p* = 0.086**	**1**	**LR = 17.76** **df = 15** ***p* = 0.275**	*M = 0.00* *(SD = 0.13)* *R: −0.24–0.18 ^e^*
	Accomplishment	4 ^a^	**0**		**0**	**0.666**	**>0.99**	0 ^d^		**1**		**1**		
	Appearance	**5**	**0**		**0**	*0.048 ^c^*	*0.0403*	0 ^d^		**1**		**1**		
	Self-Esteem	**5**	1		**0**	**0.206**	**0.086**	**1**		**1**		**1**		
	Future	5	**0**		**0**	**>0.99**	**>0.99**	**1**		**1**		**1**		
Autonomy & Daily life (7 items)	Daily Independence	**5**	2	**LR = 96.12** **df = 191** ***p* > 0.99**	**0**	**0.651**	**>0.99**	0 ^d^	*LR = 10.92* *df = 11* *p = 0.450*	**1**	**LR = 15.74** **df = 11** ***p* = 0.151**	**1**	**LR = 10.13** **df = 11** ***p* = 0.519**	*M = 0.00* *(SD = 0.12)* *R: −0.22–0.17 ^e^*
	Getting out and About	**5**	**0**		**0**	**0.86**	**>0.99**	**1**		**1**		**1**		
	Manage at School	**5**	**0**		**0**	**0.651**	**>0.99**	0 ^d^		**1**		**1**		
	Social Activities	**5**	1		**0**	**0.902**	**>0.99**	**1**		**1**		**1**		
	Decision Making	**5**	1		**0**	**>0.99**	**>0.99**	**1**		**1**		**1**		
	Support from Others	**5**	1		**0**	**>0.99**	**>0.99**	0 ^d^		**1**		**1**		
	Ability to Move	**5**	1		**0**	**0.651**	**>0.99**	0 ^d^		**1**		**1**		
Social (6 items)	Open up to Others	**5**	**0**	**LR = 79.45** **df = 143** **>0.99**	**0**	**>0.99**	**>0.99**	**1**	*LR = 11.91* *df = 11* *p = 0.370*	0 ^d^	*n.a.*	0 ^d^	*n.a.*	*M = 0.00* *(SD = 0.12)* *R: −0.19–0.25 ^e^*
	Family Relationship	**5**	1		**0**	**0.059**	*0.0423*	**1**		0 ^d^		0 ^d^		
	Relationship with Friends	**5**	**0**		**0**	**0.617**	**0.37**	0 ^d^		0 ^d^		0 ^d^		
	Friendships	**5**	1		**0**	**0.526**	**0.37**	0 ^d^		0 ^d^		0 ^d^		
	Attitudes of Others	**5**	**0**		**0**	**0.366**	**0.37**	0 ^d^		0 ^d^		0 ^d^		
	Demands from Others	**5**	1		**0**	**0.366**	**0.37**	**1**		0 ^d^		0 ^d^		
Emotions (4 items)	Loneliness	**5**	**0**	LR = 93.31 df = 63 *p* = 0.008	**0**	**0.832**	**>0.99**	**1**	**LR = 14.70** **df = 15** ***p* = 0.473**	**1**	**LR = 13.23** **df = 15** ***p* = 0.585**	**1**	**LR = 14.20** **df = 15** ***p* = 0.511**	**M = 0.00** **(SD = 0.11)** **−0.14–0.20**
	Anxiety	**5**	1		**0**	**0.832**	**>0.99**	**1**		**1**		**1**		
	Sadness	**5**	**0**		**0**	**0.832**	**>0.99**	**1**		**1**		**1**		
	Anger	**5**	**0**		*0.08 ^b^*	**0.832**	**>0.99**	**1**		**1**		**1**		
Physical (6 items)	Clumsiness	**5**	**0**	LR = 177.27 df = 143 *p* = 0.027	**0**	**>0.99**	**0.703**	**1**	**LR = 27.85** **df = 23** ***p* = 0.222**	**1**	LR = 59.53 df = 23 *p* < 0.001	**1**	**LR = 18.09** **df = 23** ***p* = 0.752**	*M = 0.00* *(SD = 0.16)* *R: −0.21–0.29 e*
	Other Injuries	**5**	2		**0**	**>0.99**	**>0.99**	**1**		**1**		**1**		
	Headaches	**5**	**0**		**0**	**>0.99**	**>0.99**	**1**		**1**		**1**		
	Pain	**5**	1		**0**	**0.435**	**0.173**	**1**		**1**		**1**		
	Seeing/Hearing	**5**	1		*0.05 ^b^*	**>0.99**	**>0.99**	**1**		**1**		**1**		
	TBI effects	**5**	2		**0**	**0.256**	**0.316**	**1**		**1**		**1**		

^a^ Missing responses in the category “not at all”; ^b^ Items only partly affected: P(X ≥ 1) = 0.17 and P(X ≥ 4) = 0.17 of the item ‘Anger’ and X P(X ≥ 2) = 0.17 of the item ‘Seeing/Hearing’; ^c^ Significant but still in acceptable range (<2.00); ^d^ Items dropped from analyses due to missing response categories either in both or in one age, TBI severity, or study setting group; ^e^ Residual correlations between the following items slightly exceed 0.20 (not exceeding 0.30). Self scale: ‘Appearance’ and ‘Self-Esteem’ (−0.23); Autonomy and Daily Life: ‘Getting out and About’ and ‘Social Activities’ (−0.22); Social scale: ‘Attitudes of Others’ and ‘Demands from Others’ (0.25); Physical scale: ‘TBI effects’ and ‘Pain’ (0.21), ‘TBI effects’ and ‘Clumsiness’ (0.21), ‘Clumsiness’ and ‘Seeing/Hearing’ (0.27), and ‘TBI effects’ and ‘Other Injuries’ (0.29). Note. **Bold** values indicate a good fit, *italic* values indicate an acceptable fit with some restrictions (e.g., dropped items or slightly higher values in some analyses).

## Data Availability

The data presented in this study are available on request from the corresponding authors. Data are not publicly available due to data protection reasons.

## Supplementary Materials

The following supporting information can be downloaded at: https://www.mdpi.com/article/10.3390/jcm12113716/s1, Online Supplement S1—Category Characteristic Curves.

## Appendix A. QOLIBRI-KID/ADO Items

**Table A1:** QOLIBRI-KID/ADO: scales and items.

Part	Type	Scale Label	Scale Title	Item #	Item Label	Item Text (Original: German)	Item Text (English) *	Item Abbreviation
1	S	A	Cognition	1	A1	wie Du Dich in der Schule konzentrieren kannst?	how you are able to concentrate at school?	Concentration
1	S	A	Cognition	2	A2	wie Du mit anderen reden kannst?	how you can talk with others?	Talking to Others
1	S	A	Cognition	3	A3	wie Du Dich an etwas erinnern kannst (zum Beispiel, was Du im Unterricht gemacht hast)?	how you are able to remember things (for example, what you did in class)?	Remembering
1	S	A	Cognition	4	A4	wie Du Deine Hausaufgaben und Freizeit (zum Beispiel Sport) planst?	how you plan your homework and leisure time (for example, sports)?	Planning
1	S	A	Cognition	5	A5	wie Du Dich zwischen zwei Dingen entscheiden kannst, wenn Du auswählen musst?	how you are able to decide between two things when you have to choose?	Decision between Two
1	S	A	Cognition	6	A6	wie Du Dich zurechtfindest (zum Beispiel den Weg zur Schule findest)?	how you are able to find your way around (for example, finding your way to school)?	Orientation
1	S	A	Cognition	7	A7	wie schnell Du denken kannst (zum Beispiel, wie lange Du nachdenken musst, um eine Antwort zu geben)?	how fast you are able to think (for example, how long you have to think to give an answer)?	Thinking Speed
1	S	B	Self	8	B1	mit Deiner Energie (zum Beispiel, wie Du in der letzten Schulstunde mitmachen kannst)?	with your energy (for example, how you are able to participate in the last lesson of the day)?	Energy
1	S	B	Self	9	B2	damit, wie Du etwas hinbekommst?	with how you manage to do something?	Accomplishment
1	S	B	Self	10	B3	damit, wie Du aussiehst?	with the way you look?	Appearance
1	S	B	Self	11	B4	mit Dir, so wie Du bist?	with yourself as you are?	Self-Esteem
1	S	B	Self	12	B5	damit, wie Du Dir Deine Zukunft vorstellst?	with how you imagine your future?	Future
1	S	C	Autonomy & Daily Life	13	C1	ohne Hilfe Dinge zu tun, die Du jeden Tag machst (zum Beispiel Dich anzuziehen)?	being able to do things you do every day without help (for example, getting dressed)?	Daily Independence
1	S	C	Autonomy & Daily Life	14	C2	wie Du draußen unterwegs sein kannst?	how you are able to be out and about?	Getting out and About
1	S	C	Autonomy & Daily Life	15	C3	wie Du in der Schule zurechtkommst?	how you manage at school?	Manage at school
1	S	C	Autonomy & Daily Life	16	C4	wie Du mit Deinen Freunden spielen/etwas unternehmen kannst?	how you are able to play/do things with your friends?	Social Activities
1	S	C	Autonomy & Daily Life	17	C5	zu entscheiden, was Du nach dem Schultag machen möchtest?	deciding what you want to do after the school day is over?	Decision Making
1	S	C	Autonomy & Daily Life	18	C6	wie andere Dich unterstützen oder Dir helfen?	how others support or help you?	Support from Others
1	S	C	Autonomy & Daily Life	19	C7	wie Du Dich bewegen kannst (zum Beispiel zu gehen, zu rennen, mit dem Rollstuhl zu fahren)?	how you are able to move around (for example, walk, run, use a wheelchair)?	Ability to Move
1	S	D	Social Relationships	20	D1	wie Du Dich anderen anvertrauen kannst?	how you are able to confide in others?	Open up to Others
1	S	D	Social Relationships	21	D2	wie Du Dich mit Deiner Familie verträgst?	how you get along with your family?	Family Relationship
1	S	D	Social Relationships	22	D3	wie Du Dich mit Deinen Freunden verträgst?	how you get along with your friends?	Relationship with Friends
1	S	D	Social Relationships	23	D4	wie Du mit jemandem befreundet bleiben kannst?	how you are able to stay friends with someone?	Friendships
1	S	D	Social Relationships	24	D5	wie andere Dich behandeln?	how others treat you?	Attitudes of Others
1	S	D	Social Relationships	25	D6	was andere von Dir verlangen?	what others demand of you?	Demands from Others
2	B	E	Emotions	26	E1	Dich einsam zu fühlen, selbst wenn andere bei Dir sind?	feeling lonely even though you are with other people?	Loneliness
2	B	E	Emotions	27	E2	ängstlich zu sein?	feeling anxious?	Anxiety
2	B	E	Emotions	28	E3	traurig zu sein?	feeling sad?	Sadness
2	B	E	Emotions	29	E4	wütend zu werden?	getting angry?	Anger
2	B	F	Physical Problems	30	F1	wenn Du ungeschickt bist (zum Beispiel, wenn Du stolperst, Dir etwas runterfällt)?	being clumsy (for example, when you trip or drop something)?	Clumsiness
2	B	F	Physical Problems	31	F2	andere Verletzungen, die Du gleichzeitig bei Deinem Unfall/Deiner Hirnverletzung abbekommen hast?	other injuries you got at the same time as your accident/brain injury?	Other Injuries
2	B	F	Physical Problems	32	F3	Kopfschmerzen?	headaches?	Headaches
2	B	F	Physical Problems	33	F4	andere Schmerzen (außer Kopfschmerzen)?	other pain (apart from headaches)?	Pain
2	B	F	Physical Problems	34	F5	Probleme beim Sehen/Hören?	problems seeing/hearing?	Seeing/Hearing
2	B	F	Physical Problems	35	F6	Veränderungen in Deinem Leben nach Deinem Unfall/Deiner Hirnverletzung?	changes in your life after your accident/brain injury?	TBI Effects

* Note that the English translation is only for understanding the meaning of the original German items and should not yet be used for data collection. Note. S: satisfaction (‘How satisfied are you with…?’), B: feeling of being bothered (‘How bothered are you with…?’).

## Appendix B. Response Behavior

**Table A2:** Response behavior for the Cognition scale stratified by age group, TBI severity, and study setting.

Cognition		Age Group		TBI Severity			Study Setting		
		Children	Adolescents	Mild	Moderate	Severe	Offline	Online	Total
Item	Response	(N = 152)	(N = 148)	(N = 215)	(N = 25)	(N = 60)	(N = 224)	(N = 76)	(N = 300)
Concentration	not at all	**0 (0%)**	5 (3.4%)	0 (0%)	1 (4.0%)	4 (6.7%)	4 (1.8%)	1 (1.3%)	5 (1.7%)
	slightly	3 (2.0%)	9 (6.1%)	9 (4.2%)	0 (0%)	3 (5.0%)	9 (4.0%)	3 (3.9%)	12 (4.0%)
	moderately	44 (28.9%)	41 (27.7%)	60 (27.9%)	5 (20.0%)	20 (33.3%)	62 (27.7%)	23 (30.3%)	85 (28.3%)
	quite	70 (46.1%)	69 (46.6%)	100 (46.5%)	13 (52.0%)	26 (43.3%)	105 (46.9%)	34 (44.7%)	139 (46.3%)
	very	35 (23.0%)	19 (12.8%)	41 (19.1%)	6 (24.0%)	7 (11.7%)	40 (17.9%)	14 (18.4%)	54 (18.0%)
	missing	0 (0%)	5 (3.4%)	5 (2.3%)	0 (0%)	0 (0%)	4 (1.8%)	1 (1.3%)	5 (1.7%)
Talking to Others	not at all	**0 (0%)**	3 (2.0%)	1 (0.5%)	0 (0%)	2 (3.3%)	2 (0.9%)	1 (1.3%)	3 (1.0%)
	slightly	1 (0.7%)	5 (3.4%)	3 (1.4%)	1 (4.0%)	2 (3.3%)	5 (2.2%)	1 (1.3%)	6 (2.0%)
	moderately	11 (7.2%)	18 (12.2%)	23 (10.7%)	1 (4.0%)	5 (8.3%)	24 (10.7%)	5 (6.6%)	29 (9.7%)
	quite	40 (26.3%)	56 (37.8%)	77 (35.8%)	5 (20.0%)	14 (23.3%)	66 (29.5%)	30 (39.5%)	96 (32.0%)
	very	100 (65.8%)	66 (44.6%)	111 (51.6%)	18 (72.0%)	37 (61.7%)	127 (56.7%)	39 (51.3%)	166 (55.3%)
	missing	0 (0%)	0 (0%)	0 (0%)	0 (0%)	0 (0%)	0 (0%)	0 (0%)	0 (0%)
Remembering	not at all	3 (2.0%)	5 (3.4%)	5 (2.3%)	**0 (0%)**	3 (5.0%)	7 (3.1%)	1 (1.3%)	8 (2.7%)
	slightly	5 (3.3%)	12 (8.1%)	10 (4.7%)	1 (4.0%)	6 (10.0%)	13 (5.8%)	4 (5.3%)	17 (5.7%)
	moderately	36 (23.7%)	43 (29.1%)	56 (26.0%)	5 (20.0%)	18 (30.0%)	55 (24.6%)	24 (31.6%)	79 (26.3%)
	quite	50 (32.9%)	52 (35.1%)	75 (34.9%)	11 (44.0%)	16 (26.7%)	75 (33.5%)	27 (35.5%)	102 (34.0%)
	very	58 (38.2%)	33 (22.3%)	67 (31.2%)	8 (32.0%)	16 (26.7%)	71 (31.7%)	20 (26.3%)	91 (30.3%)
	missing	0 (0%)	3 (2.0%)	2 (0.9%)	0 (0%)	1 (1.7%)	3 (1.3%)	0 (0%)	3 (1.0%)
Planning	not at all	1 (0.7%)	9 (6.1%)	5 (2.3%)	1 (4.0%)	4 (6.7%)	8 (3.6%)	2 (2.6%)	10 (3.3%)
	slightly	3 (2.0%)	10 (6.8%)	11 (5.1%)	1 (4.0%)	1 (1.7%)	10 (4.5%)	3 (3.9%)	13 (4.3%)
	moderately	24 (15.8%)	45 (30.4%)	51 (23.7%)	2 (8.0%)	16 (26.7%)	54 (24.1%)	15 (19.7%)	69 (23.0%)
	quite	51 (33.6%)	49 (33.1%)	77 (35.8%)	8 (32.0%)	15 (25.0%)	67 (29.9%)	33 (43.4%)	100 (33.3%)
	very	73 (48.0%)	34 (23.0%)	71 (33.0%)	13 (52.0%)	23 (38.3%)	84 (37.5%)	23 (30.3%)	107 (35.7%)
	missing	0 (0%)	1 (0.7%)	0 (0%)	0 (0%)	1 (1.7%)	1 (0.4%)	0 (0%)	1 (0.3%)
Decision Between Two	not at all	8 (5.3%)	8 (5.4%)	11 (5.1%)	**0 (0%)**	5 (8.3%)	12 (5.4%)	4 (5.3%)	16 (5.3%)
	slightly	10 (6.6%)	15 (10.1%)	17 (7.9%)	3 (12.0%)	5 (8.3%)	17 (7.6%)	8 (10.5%)	25 (8.3%)
	moderately	55 (36.2%)	49 (33.1%)	73 (34.0%)	8 (32.0%)	23 (38.3%)	79 (35.3%)	25 (32.9%)	104 (34.7%)
	quite	60 (39.5%)	57 (38.5%)	90 (41.9%)	8 (32.0%)	19 (31.7%)	83 (37.1%)	34 (44.7%)	117 (39.0%)
	very	19 (12.5%)	19 (12.8%)	24 (11.2%)	6 (24.0%)	8 (13.3%)	33 (14.7%)	5 (6.6%)	38 (12.7%)
	missing	0 (0%)	0 (0%)	0 (0%)	0 (0%)	0 (0%)	0 (0%)	0 (0%)	0 (0%)
Orientation	not at all	**0 (0%)**	**0 (0%)**	**0 (0%)**	**0 (0%)**	**0 (0%)**	**0 (0%)**	**0 (0%)**	**0 (0%)**
	slightly	1 (0.7%)	0 (0%)	0 (0%)	0 (0%)	1 (1.7%)	1 (0.4%)	0 (0%)	1 (0.3%)
	moderately	8 (5.3%)	8 (5.4%)	7 (3.3%)	2 (8.0%)	7 (11.7%)	10 (4.5%)	6 (7.9%)	16 (5.3%)
	quite	21 (13.8%)	24 (16.2%)	25 (11.6%)	5 (20.0%)	15 (25.0%)	34 (15.2%)	11 (14.5%)	45 (15.0%)
	very	122 (80.3%)	116 (78.4%)	183 (85.1%)	18 (72.0%)	37 (61.7%)	179 (79.9%)	59 (77.6%)	238 (79.3%)
	missing	0 (0%)	0 (0%)	0 (0%)	0 (0%)	0 (0%)	0 (0%)	0 (0%)	0 (0%)
Thinking Speed	not at all	2 (1.3%)	1 (0.7%)	1 (0.5%)	**0 (0%)**	2 (3.3%)	2 (0.9%)	1 (1.3%)	3 (1.0%)
	slightly	8 (5.3%)	11 (7.4%)	11 (5.1%)	1 (4.0%)	7 (11.7%)	13 (5.8%)	6 (7.9%)	19 (6.3%)
	moderately	24 (15.8%)	34 (23.0%)	40 (18.6%)	4 (16.0%)	14 (23.3%)	46 (20.5%)	12 (15.8%)	58 (19.3%)
	quite	64 (42.1%)	68 (45.9%)	99 (46.0%)	11 (44.0%)	22 (36.7%)	102 (45.5%)	30 (39.5%)	132 (44.0%)
	very	54 (35.5%)	34 (23.0%)	64 (29.8%)	9 (36.0%)	15 (25.0%)	61 (27.2%)	27 (35.5%)	88 (29.3%)
	missing	0 (0%)	0 (0%)	0 (0%)	0 (0%)	0 (0%)	0 (0%)	0 (0%)	0 (0%)

Note. Study setting: face-to-face interviews conducted either offline or online (via video call). **Bold** values indicate response categories with zero endorsements.

**Table A3:** Response behavior for the Self scale stratified by age group, TBI severity, and study setting.

Self		Age Group		TBI Severity			Study Setting		
		Children	Adolescents	Mild	Moderate	Severe	Offline	Online	Total
Item	Response	(N = 152)	(N = 148)	(N = 215)	(N = 25)	(N = 60)	(N = 224)	(N = 76)	(N = 300)
Energy	not at all	1 (0.7%)	2 (1.4%)	2 (0.9%)	0 (0%)	1 (1.7%)	2 (0.9%)	1 (1.3%)	3 (1.0%)
	slightly	3 (2.0%)	17 (11.5%)	16 (7.4%)	1 (4.0%)	3 (5.0%)	15 (6.7%)	5 (6.6%)	20 (6.7%)
	moderately	21 (13.8%)	45 (30.4%)	51 (23.7%)	4 (16.0%)	11 (18.3%)	45 (20.1%)	21 (27.6%)	66 (22.0%)
	quite	53 (34.9%)	52 (35.1%)	73 (34.0%)	9 (36.0%)	23 (38.3%)	82 (36.6%)	23 (30.3%)	105 (35.0%)
	very	74 (48.7%)	29 (19.6%)	70 (32.6%)	11 (44.0%)	22 (36.7%)	77 (34.4%)	26 (34.2%)	103 (34.3%)
	missing	0 (0%)	3 (2.0%)	3 (1.4%)	0 (0%)	0 (0%)	3 (1.3%)	0 (0%)	3 (1.0%)
Accomplishment	not at all	**0 (0%)**	**0 (0%)**	**0 (0%)**	**0 (0%)**	**0 (0%)**	**0 (0%)**	**0 (0%)**	**0 (0%)**
	slightly	**0 (0%)**	2 (1.4%)	1 (0.5%)	0 (0%)	1 (1.7%)	0 (0%)	2 (2.6%)	2 (0.7%)
	moderately	3 (2.0%)	7 (4.7%)	7 (3.3%)	1 (4.0%)	2 (3.3%)	5 (2.2%)	5 (6.6%)	10 (3.3%)
	quite	39 (25.7%)	58 (39.2%)	65 (30.2%)	6 (24.0%)	26 (43.3%)	72 (32.1%)	25 (32.9%)	97 (32.3%)
	very	110 (72.4%)	81 (54.7%)	142 (66.0%)	18 (72.0%)	31 (51.7%)	147 (65.6%)	44 (57.9%)	191 (63.7%)
	missing	0 (0%)	0 (0%)	0 (0%)	0 (0%)	0 (0%)	0 (0%)	0 (0%)	0 (0%)
Appearance	not at all	**0 (0%)**	3 (2.0%)	2 (0.9%)	**0 (0%)**	1 (1.7%)	2 (0.9%)	1 (1.3%)	3 (1.0%)
	slightly	2 (1.3%)	8 (5.4%)	6 (2.8%)	**0 (0%)**	4 (6.7%)	6 (2.7%)	4 (5.3%)	10 (3.3%)
	moderately	21 (13.8%)	44 (29.7%)	53 (24.7%)	5 (20.0%)	7 (11.7%)	48 (21.4%)	17 (22.4%)	65 (21.7%)
	quite	55 (36.2%)	65 (43.9%)	88 (40.9%)	10 (40.0%)	22 (36.7%)	86 (38.4%)	34 (44.7%)	120 (40.0%)
	very	74 (48.7%)	27 (18.2%)	66 (30.7%)	10 (40.0%)	25 (41.7%)	81 (36.2%)	20 (26.3%)	101 (33.7%)
	missing	0 (0%)	1 (0.7%)	0 (0%)	0 (0%)	1 (1.7%)	1 (0.4%)	0 (0%)	1 (0.3%)
Self-Esteem	not at all	1 (0.7%)	3 (2.0%)	2 (0.9%)	**0 (0%)**	2 (3.3%)	1 (0.4%)	3 (3.9%)	4 (1.3%)
	slightly	1 (0.7%)	7 (4.7%)	7 (3.3%)	**0 (0%)**	1 (1.7%)	4 (1.8%)	4 (5.3%)	8 (2.7%)
	moderately	17 (11.2%)	32 (21.6%)	34 (15.8%)	2 (8.0%)	13 (21.7%)	40 (17.9%)	9 (11.8%)	49 (16.3%)
	quite	43 (28.3%)	58 (39.2%)	80 (37.2%)	7 (28.0%)	14 (23.3%)	72 (32.1%)	29 (38.2%)	101 (33.7%)
	very	90 (59.2%)	47 (31.8%)	91 (42.3%)	16 (64.0%)	30 (50.0%)	106 (47.3%)	31 (40.8%)	137 (45.7%)
	missing	0 (0%)	1 (0.7%)	1 (0.5%)	0 (0%)	0 (0%)	1 (0.4%)	0 (0%)	1 (0.3%)
Future	not at all	**0 (0%)**	3 (2.0%)	1 (0.5%)	**0 (0%)**	2 (3.3%)	2 (0.9%)	1 (1.3%)	3 (1.0%)
	slightly	1 (0.7%)	10 (6.8%)	6 (2.8%)	**0 (0%)**	5 (8.3%)	7 (3.1%)	4 (5.3%)	11 (3.7%)
	moderately	11 (7.2%)	27 (18.2%)	30 (14.0%)	**0 (0%)**	8 (13.3%)	28 (12.5%)	10 (13.2%)	38 (12.7%)
	quite	50 (32.9%)	54 (36.5%)	72 (33.5%)	13 (52.0%)	19 (31.7%)	75 (33.5%)	29 (38.2%)	104 (34.7%)
	very	89 (58.6%)	53 (35.8%)	104 (48.4%)	12 (48.0%)	26 (43.3%)	110 (49.1%)	32 (42.1%)	142 (47.3%)
	missing	1 (0.7%)	1 (0.7%)	2 (0.9%)	0 (0%)	0 (0%)	2 (0.9%)	0 (0%)	2 (0.7%)

Note. Study setting: Face-to-face interviews conducted either offline or online (via video call). **Bold** values indicate response categories with zero endorsements.

**Table A4:** Response behavior for the Autonomy & Daily Life scale stratified by age group, TBI severity, and study setting.

Autonomy & Daily Life		Age Group		TBI Severity			Study Setting		
		Children	Adolescents	Mild	Moderate	Severe	Offline	Online	Total
Item	Response	(N = 152)	(N = 148)	(N = 215)	(N = 25)	(N = 60)	(N = 224)	(N = 76)	(N = 300)
Daily Independence	not at all	**0 (0%)**	1 (0.7%)	1 (0.5%)	**0 (0%)**	**0 (0%)**	1 (0.4%)	**0 (0%)**	1 (0.3%)
	slightly	1 (0.7%)	1 (0.7%)	2 (0.9%)	**0 (0%)**	**0 (0%)**	2 (0.9%)	**0 (0%)**	2 (0.7%)
	moderately	5 (3.3%)	1 (0.7%)	4 (1.9%)	2 (8.0%)	**0 (0%)**	4 (1.8%)	2 (2.6%)	6 (2.0%)
	quite	19 (12.5%)	9 (6.1%)	15 (7.0%)	4 (16.0%)	9 (15.0%)	19 (8.5%)	9 (11.8%)	28 (9.3%)
	very	124 (81.6%)	133 (89.9%)	189 (87.9%)	19 (76.0%)	49 (81.7%)	193 (86.2%)	64 (84.2%)	257 (85.7%)
	missing	3 (2.0%)	3 (2.0%)	4 (1.9%)	0 (0%)	2 (3.3%)	5 (2.2%)	1 (1.3%)	6 (2.0%)
Getting out and About	not at all	1 (0.7%)	2 (1.4%)	2 (0.9%)	**0 (0%)**	1 (1.7%)	3 (1.3%)	**0 (0%)**	3 (1.0%)
	slightly	5 (3.3%)	4 (2.7%)	7 (3.3%)	1 (4.0%)	1 (1.7%)	7 (3.1%)	2 (2.6%)	9 (3.0%)
	moderately	9 (5.9%)	16 (10.8%)	19 (8.8%)	1 (4.0%)	5 (8.3%)	20 (8.9%)	5 (6.6%)	25 (8.3%)
	quite	25 (16.4%)	27 (18.2%)	38 (17.7%)	5 (20.0%)	9 (15.0%)	33 (14.7%)	19 (25.0%)	52 (17.3%)
	very	111 (73.0%)	96 (64.9%)	145 (67.4%)	18 (72.0%)	44 (73.3%)	157 (70.1%)	50 (65.8%)	207 (69.0%)
	missing	1 (0.7%)	3 (2.0%)	4 (1.9%)	0 (0%)	0 (0%)	4 (1.8%)	0 (0%)	4 (1.3%)
Manage at School	not at all	**0 (0%)**	3 (2.0%)	**0 (0%)**	**0 (0%)**	3 (5.0%)	2 (0.9%)	1 (1.3%)	3 (1.0%)
	slightly	6 (3.9%)	6 (4.1%)	8 (3.7%)	**0 (0%)**	4 (6.7%)	9 (4.0%)	3 (3.9%)	12 (4.0%)
	moderately	19 (12.5%)	28 (18.9%)	32 (14.9%)	3 (12.0%)	12 (20.0%)	35 (15.6%)	12 (15.8%)	47 (15.7%)
	quite	59 (38.8%)	72 (48.6%)	105 (48.8%)	6 (24.0%)	20 (33.3%)	98 (43.8%)	33 (43.4%)	131 (43.7%)
	very	68 (44.7%)	34 (23.0%)	65 (30.2%)	16 (64.0%)	21 (35.0%)	76 (33.9%)	26 (34.2%)	102 (34.0%)
	missing	0 (0%)	5 (3.4%)	5 (2.3%)	0 (0%)	0 (0%)	4 (1.8%)	1 (1.3%)	5 (1.7%)
Social Activities	not at all	3 (2.0%)	3 (2.0%)	1 (0.5%)	1 (4.0%)	4 (6.7%)	5 (2.2%)	1 (1.3%)	6 (2.0%)
	slightly	1 (0.7%)	4 (2.7%)	3 (1.4%)	**0 (0%)**	2 (3.3%)	4 (1.8%)	1 (1.3%)	5 (1.7%)
	moderately	6 (3.9%)	19 (12.8%)	17 (7.9%)	**0 (0%)**	8 (13.3%)	17 (7.6%)	8 (10.5%)	25 (8.3%)
	quite	37 (24.3%)	44 (29.7%)	67 (31.2%)	4 (16.0%)	10 (16.7%)	58 (25.9%)	23 (30.3%)	81 (27.0%)
	very	105 (69.1%)	76 (51.4%)	126 (58.6%)	20 (80.0%)	35 (58.3%)	138 (61.6%)	43 (56.6%)	181 (60.3%)
	missing	0 (0%)	2 (1.4%)	1 (0.5%)	0 (0%)	1 (1.7%)	2 (0.9%)	0 (0%)	2 (0.7%)
Decision Making	not at all	1 (0.7%)	2 (1.4%)	1 (0.5%)	**0 (0%)**	2 (3.3%)	2 (0.9%)	1 (1.3%)	3 (1.0%)
	slightly	1 (0.7%)	2 (1.4%)	2 (0.9%)	**0 (0%)**	1 (1.7%)	2 (0.9%)	1 (1.3%)	3 (1.0%)
	moderately	14 (9.2%)	20 (13.5%)	27 (12.6%)	1 (4.0%)	6 (10.0%)	22 (9.8%)	12 (15.8%)	34 (11.3%)
	quite	57 (37.5%)	59 (39.9%)	79 (36.7%)	12 (48.0%)	25 (41.7%)	89 (39.7%)	27 (35.5%)	116 (38.7%)
	very	79 (52.0%)	61 (41.2%)	103 (47.9%)	12 (48.0%)	25 (41.7%)	106 (47.3%)	34 (44.7%)	140 (46.7%)
	missing	0 (0%)	4 (2.7%)	3 (1.4%)	0 (0%)	1 (1.7%)	3 (1.3%)	1 (1.3%)	4 (1.3%)
Support from Others	not at all	1 (0.7%)	**0 (0%)**	**0 (0%)**	1 (4.0%)	**0 (0%)**	1 (0.4%)	**0 (0%)**	1 (0.3%)
	slightly	1 (0.7%)	1 (0.7%)	2 (0.9%)	**0 (0%)**	**0 (0%)**	2 (0.9%)	**0 (0%)**	2 (0.7%)
	moderately	7 (4.6%)	16 (10.8%)	14 (6.5%)	1 (4.0%)	8 (13.3%)	15 (6.7%)	8 (10.5%)	23 (7.7%)
	quite	48 (31.6%)	63 (42.6%)	81 (37.7%)	7 (28.0%)	23 (38.3%)	82 (36.6%)	29 (38.2%)	111 (37.0%)
	very	95 (62.5%)	66 (44.6%)	117 (54.4%)	16 (64.0%)	28 (46.7%)	122 (54.5%)	39 (51.3%)	161 (53.7%)
	missing	0 (0%)	2 (1.4%)	1 (0.5%)	0 (0%)	1 (1.7%)	2 (0.9%)	0 (0%)	2 (0.7%)
Ability to Move	not at all	**0 (0%)**	1 (0.7%)	1 (0.5%)	**0 (0%)**	**0 (0%)**	1 (0.4%)	**0 (0%)**	1 (0.3%)
	slightly	3 (2.0%)	3 (2.0%)	2 (0.9%)	1 (4.0%)	3 (5.0%)	5 (2.2%)	1 (1.3%)	6 (2.0%)
	moderately	5 (3.3%)	11 (7.4%)	12 (5.6%)	0 (0%)	4 (6.7%)	9 (4.0%)	7 (9.2%)	16 (5.3%)
	quite	25 (16.4%)	25 (16.9%)	38 (17.7%)	2 (8.0%)	10 (16.7%)	37 (16.5%)	13 (17.1%)	50 (16.7%)
	very	118 (77.6%)	107 (72.3%)	160 (74.4%)	22 (88.0%)	43 (71.7%)	170 (75.9%)	55 (72.4%)	225 (75.0%)
	missing	1 (0.7%)	1 (0.7%)	2 (0.9%)	0 (0%)	0 (0%)	2 (0.9%)	0 (0%)	2 (0.7%)

Note. Study setting: face-to-face interviews conducted either offline or online (via video call). **Bold** values indicate response categories with zero endorsements.

**Table A5:** Response behavior for the Social scale stratified by age group, TBI severity, and study setting.

Social		Age Group		TBI Severity			Study Setting		
		Children	Adolescents	Mild	Moderate	Severe	Offline	Online	Total
Item	Response	(N = 152)	(N = 148)	(N = 215)	(N = 25)	(N = 60)	(N = 224)	(N = 76)	(N = 300)
Open up to Others	not at all	1 (0.7%)	1 (0.7%)	1 (0.5%)	**0 (0%)**	1 (1.7%)	1 (0.4%)	1 (1.3%)	2 (0.7%)
	slightly	2 (1.3%)	13 (8.8%)	13 (6.0%)	1 (4.0%)	1 (1.7%)	14 (6.3%)	1 (1.3%)	15 (5.0%)
	moderately	11 (7.2%)	28 (18.9%)	29 (13.5%)	2 (8.0%)	8 (13.3%)	28 (12.5%)	11 (14.5%)	39 (13.0%)
	quite	69 (45.4%)	54 (36.5%)	83 (38.6%)	11 (44.0%)	29 (48.3%)	83 (37.1%)	40 (52.6%)	123 (41.0%)
	very	69 (45.4%)	50 (33.8%)	88 (40.9%)	11 (44.0%)	20 (33.3%)	96 (42.9%)	23 (30.3%)	119 (39.7%)
	missing	0 (0%)	2 (1.4%)	1 (0.5%)	0 (0%)	1 (1.7%)	2 (0.9%)	0 (0%)	2 (0.7%)
Family Relationship	not at all	1 (0.7%)	2 (1.4%)	2 (0.9%)	**0 (0%)**	1 (1.7%)	3 (1.3%)	**0 (0%)**	3 (1.0%)
	slightly	2 (1.3%)	2 (1.4%)	4 (1.9%)	**0 (0%)**	**0 (0%)**	3 (1.3%)	1 (1.3%)	4 (1.3%)
	moderately	25 (16.4%)	25 (16.9%)	34 (15.8%)	2 (8.0%)	14 (23.3%)	36 (16.1%)	14 (18.4%)	50 (16.7%)
	quite	57 (37.5%)	67 (45.3%)	102 (47.4%)	9 (36.0%)	13 (21.7%)	93 (41.5%)	31 (40.8%)	124 (41.3%)
	very	67 (44.1%)	50 (33.8%)	72 (33.5%)	14 (56.0%)	31 (51.7%)	87 (38.8%)	30 (39.5%)	117 (39.0%)
	missing	0 (0%)	2 (1.4%)	1 (0.5%)	0 (0%)	1 (1.7%)	2 (0.9%)	0 (0%)	2 (0.7%)
Relationship with Friends	not at all	**0 (0%)**	1 (0.7%)	**0 (0%)**	**0 (0%)**	1 (1.7%)	**0 (0%)**	1 (1.3%)	1 (0.3%)
	slightly	2 (1.3%)	1 (0.7%)	2 (0.9%)	1 (4.0%)	**0 (0%)**	3 (1.3%)	**0 (0%)**	3 (1.0%)
	moderately	9 (5.9%)	5 (3.4%)	9 (4.2%)	0 (0%)	5 (8.3%)	8 (3.6%)	6 (7.9%)	14 (4.7%)
	quite	37 (24.3%)	53 (35.8%)	70 (32.6%)	6 (24.0%)	14 (23.3%)	70 (31.3%)	20 (26.3%)	90 (30.0%)
	very	104 (68.4%)	88 (59.5%)	134 (62.3%)	18 (72.0%)	40 (66.7%)	143 (63.8%)	49 (64.5%)	192 (64.0%)
	missing	0 (0%)	0 (0%)	0 (0%)	0 (0%)	0 (0%)	0 (0%)	0 (0%)	0 (0%)
Friendships	not at all	**0 (0%)**	1 (0.7%)	**0 (0%)**	**0 (0%)**	1 (1.7%)	**0 (0%)**	1 (1.3%)	1 (0.3%)
	slightly	2 (1.3%)	3 (2.0%)	2 (0.9%)	1 (4.0%)	2 (3.3%)	5 (2.2%)	**0 (0%)**	5 (1.7%)
	moderately	4 (2.6%)	9 (6.1%)	11 (5.1%)	1 (4.0%)	1 (1.7%)	9 (4.0%)	4 (5.3%)	13 (4.3%)
	quite	28 (18.4%)	45 (30.4%)	59 (27.4%)	3 (12.0%)	11 (18.3%)	52 (23.2%)	21 (27.6%)	73 (24.3%)
	very	118 (77.6%)	90 (60.8%)	143 (66.5%)	20 (80.0%)	45 (75.0%)	158 (70.5%)	50 (65.8%)	208 (69.3%)
	missing	0 (0%)	0 (0%)	0 (0%)	0 (0%)	0 (0%)	0 (0%)	0 (0%)	0 (0%)
Attitudes of Others	not at all	**0 (0%)**	1 (0.7%)	**0 (0%)**	**0 (0%)**	1 (1.7%)	1 (0.4%)	**0 (0%)**	1 (0.3%)
	slightly	6 (3.9%)	6 (4.1%)	5 (2.3%)	2 (8.0%)	5 (8.3%)	9 (4.0%)	3 (3.9%)	12 (4.0%)
	moderately	18 (11.8%)	22 (14.9%)	25 (11.6%)	2 (8.0%)	13 (21.7%)	25 (11.2%)	15 (19.7%)	40 (13.3%)
	quite	62 (40.8%)	68 (45.9%)	98 (45.6%)	10 (40.0%)	22 (36.7%)	99 (44.2%)	31 (40.8%)	130 (43.3%)
	very	66 (43.4%)	50 (33.8%)	86 (40.0%)	11 (44.0%)	19 (31.7%)	89 (39.7%)	27 (35.5%)	116 (38.7%)
	missing	0 (0%)	1 (0.7%)	1 (0.5%)	0 (0%)	0 (0%)	1 (0.4%)	0 (0%)	1 (0.3%)
Demands from Others	not at all	1 (0.7%)	2 (1.4%)	**0 (0%)**	**0 (0%)**	3 (5.0%)	3 (1.3%)	**0 (0%)**	3 (1.0%)
	slightly	3 (2.0%)	7 (4.7%)	7 (3.3%)	1 (4.0%)	2 (3.3%)	7 (3.1%)	3 (3.9%)	10 (3.3%)
	moderately	26 (17.1%)	30 (20.3%)	40 (18.6%)	2 (8.0%)	14 (23.3%)	41 (18.3%)	15 (19.7%)	56 (18.7%)
	quite	80 (52.6%)	80 (54.1%)	116 (54.0%)	15 (60.0%)	29 (48.3%)	119 (53.1%)	41 (53.9%)	160 (53.3%)
	very	42 (27.6%)	29 (19.6%)	52 (24.2%)	7 (28.0%)	12 (20.0%)	54 (24.1%)	17 (22.4%)	71 (23.7%)
	missing	0 (0%)	0 (0%)	0 (0%)	0 (0%)	0 (0%)	0 (0%)	0 (0%)	0 (0%)

Note. Study setting: face-to-face interviews conducted either offline or online (via video call). **Bold** values indicate response categories with zero endorsements.

**Table A6:** Response behavior for the Emotions scale stratified by age group, TBI severity, and study setting.

Emotions *		Age Group		TBI Severity			Study Setting		
		Children	Adolescents	Mild	Moderate	Severe	Offline	Online	Total
Item	Response	(N = 152)	(N = 148)	(N = 215)	(N = 25)	(N = 60)	(N = 224)	(N = 76)	(N = 300)
Loneliness	very	26 (17.1%)	18 (12.2%)	33 (15.3%)	2 (8.0%)	9 (15.0%)	34 (15.2%)	10 (13.2%)	44 (14.7%)
	quite	19 (12.5%)	24 (16.2%)	35 (16.3%)	4 (16.0%)	4 (6.7%)	32 (14.3%)	11 (14.5%)	43 (14.3%)
	moderately	21 (13.8%)	28 (18.9%)	35 (16.3%)	4 (16.0%)	10 (16.7%)	38 (17.0%)	11 (14.5%)	49 (16.3%)
	slightly	33 (21.7%)	32 (21.6%)	45 (20.9%)	4 (16.0%)	16 (26.7%)	41 (18.3%)	24 (31.6%)	65 (21.7%)
	not at all	51 (33.6%)	44 (29.7%)	63 (29.3%)	11 (44.0%)	21 (35.0%)	78 (34.8%)	17 (22.4%)	95 (31.7%)
	missing	2 (1.3%)	2 (1.4%)	4 (1.9%)	0 (0%)	0 (0%)	1 (0.4%)	3 (3.9%)	4 (1.3%)
Anxiety	very	16 (10.5%)	18 (12.2%)	29 (13.5%)	1 (4.0%)	4 (6.7%)	28 (12.5%)	6 (7.9%)	34 (11.3%)
	quite	23 (15.1%)	34 (23.0%)	41 (19.1%)	5 (20.0%)	11 (18.3%)	41 (18.3%)	16 (21.1%)	57 (19.0%)
	moderately	52 (34.2%)	42 (28.4%)	62 (28.8%)	8 (32.0%)	24 (40.0%)	69 (30.8%)	25 (32.9%)	94 (31.3%)
	slightly	26 (17.1%)	30 (20.3%)	42 (19.5%)	7 (28.0%)	7 (11.7%)	43 (19.2%)	13 (17.1%)	56 (18.7%)
	not at all	35 (23.0%)	23 (15.5%)	40 (18.6%)	4 (16.0%)	14 (23.3%)	42 (18.8%)	16 (21.1%)	58 (19.3%)
	missing	0 (0%)	1 (0.7%)	1 (0.5%)	0 (0%)	0 (0%)	1 (0.4%)	0 (0%)	1 (0.3%)
Sadness	very	30 (19.7%)	18 (12.2%)	32 (14.9%)	3 (12.0%)	13 (21.7%)	40 (17.9%)	8 (10.5%)	48 (16.0%)
	quite	36 (23.7%)	41 (27.7%)	58 (27.0%)	4 (16.0%)	15 (25.0%)	56 (25.0%)	21 (27.6%)	77 (25.7%)
	moderately	38 (25.0%)	33 (22.3%)	49 (22.8%)	9 (36.0%)	13 (21.7%)	53 (23.7%)	18 (23.7%)	71 (23.7%)
	slightly	23 (15.1%)	29 (19.6%)	38 (17.7%)	4 (16.0%)	10 (16.7%)	39 (17.4%)	13 (17.1%)	52 (17.3%)
	not at all	25 (16.4%)	27 (18.2%)	38 (17.7%)	5 (20.0%)	9 (15.0%)	36 (16.1%)	16 (21.1%)	52 (17.3%)
	missing	0 (0%)	0 (0%)	0 (0%)	0 (0%)	0 (0%)	0 (0%)	0 (0%)	0 (0%)
Anger	very	25 (16.4%)	14 (9.5%)	29 (13.5%)	2 (8.0%)	8 (13.3%)	29 (12.9%)	10 (13.2%)	39 (13.0%)
	quite	31 (20.4%)	36 (24.3%)	47 (21.9%)	4 (16.0%)	16 (26.7%)	46 (20.5%)	21 (27.6%)	67 (22.3%)
	moderately	50 (32.9%)	46 (31.1%)	67 (31.2%)	12 (48.0%)	17 (28.3%)	74 (33.0%)	22 (28.9%)	96 (32.0%)
	slightly	29 (19.1%)	32 (21.6%)	50 (23.3%)	3 (12.0%)	8 (13.3%)	48 (21.4%)	13 (17.1%)	61 (20.3%)
	not at all	17 (11.2%)	20 (13.5%)	22 (10.2%)	4 (16.0%)	11 (18.3%)	27 (12.1%)	10 (13.2%)	37 (12.3%)
	missing	0 (0%)	0 (0%)	0 (0%)	0 (0%)	0 (0%)	0 (0%)	0 (0%)	0 (0%)

Note. * Negatively worded scale measuring being bothered by TBI consequences. Study setting: face-to-face interviews conducted either offline or online (via video call).

**Table A7:** Response behavior for the Physical scale stratified by age group, TBI severity, and study setting.

Physical *		Age Group		TBI Severity			Study Setting		
		Children	Adolescents	Mild	Moderate	Severe	Offline	Online	Total
Item	Response	(N = 152)	(N = 148)	(N = 215)	(N = 25)	(N = 60)	(N = 224)	(N = 76)	(N = 300)
Clumsiness	very	20 (13.2%)	16 (10.8%)	26 (12.1%)	**0 (0%)**	10 (16.7%)	27 (12.1%)	9 (11.8%)	36 (12.0%)
	quite	27 (17.8%)	29 (19.6%)	42 (19.5%)	4 (16.0%)	10 (16.7%)	42 (18.8%)	14 (18.4%)	56 (18.7%)
	moderately	44 (28.9%)	47 (31.8%)	67 (31.2%)	11 (44.0%)	13 (21.7%)	68 (30.4%)	23 (30.3%)	91 (30.3%)
	slightly	36 (23.7%)	33 (22.3%)	47 (21.9%)	6 (24.0%)	16 (26.7%)	49 (21.9%)	20 (26.3%)	69 (23.0%)
	not at all	24 (15.8%)	23 (15.5%)	32 (14.9%)	4 (16.0%)	11 (18.3%)	37 (16.5%)	10 (13.2%)	47 (15.7%)
	missing	1 (0.7%)	0 (0%)	1 (0.5%)	0 (0%)	0 (0%)	1 (0.4%)	0 (0%)	1 (0.3%)
Other Injuries	very	16 (10.5%)	8 (5.4%)	13 (6.0%)	2 (8.0%)	9 (15.0%)	16 (7.1%)	8 (10.5%)	24 (8.0%)
	quite	14 (9.2%)	12 (8.1%)	15 (7.0%)	4 (16.0%)	7 (11.7%)	20 (8.9%)	6 (7.9%)	26 (8.7%)
	moderately	23 (15.1%)	21 (14.2%)	26 (12.1%)	3 (12.0%)	15 (25.0%)	31 (13.8%)	13 (17.1%)	44 (14.7%)
	slightly	18 (11.8%)	19 (12.8%)	26 (12.1%)	4 (16.0%)	7 (11.7%)	28 (12.5%)	9 (11.8%)	37 (12.3%)
	not at all	78 (51.3%)	83 (56.1%)	127 (59.1%)	12 (48.0%)	22 (36.7%)	123 (54.9%)	38 (50.0%)	161 (53.7%)
	missing	3 (2.0%)	5 (3.4%)	8 (3.7%)	0 (0%)	0 (0%)	6 (2.7%)	2 (2.6%)	8 (2.7%)
Headaches	very	36 (23.7%)	22 (14.9%)	40 (18.6%)	4 (16.0%)	14 (23.3%)	43 (19.2%)	15 (19.7%)	58 (19.3%)
	quite	33 (21.7%)	32 (21.6%)	47 (21.9%)	5 (20.0%)	13 (21.7%)	52 (23.2%)	13 (17.1%)	65 (21.7%)
	moderately	24 (15.8%)	31 (20.9%)	40 (18.6%)	5 (20.0%)	10 (16.7%)	34 (15.2%)	21 (27.6%)	55 (18.3%)
	slightly	24 (15.8%)	30 (20.3%)	39 (18.1%)	7 (28.0%)	8 (13.3%)	42 (18.8%)	12 (15.8%)	54 (18.0%)
	not at all	35 (23.0%)	33 (22.3%)	49 (22.8%)	4 (16.0%)	15 (25.0%)	53 (23.7%)	15 (19.7%)	68 (22.7%)
	missing	0 (0%)	0 (0%)	0 (0%)	0 (0%)	0 (0%)	0 (0%)	0 (0%)	0 (0%)
Pain	very	27 (17.8%)	15 (10.1%)	31 (14.4%)	2 (8.0%)	9 (15.0%)	33 (14.7%)	9 (11.8%)	42 (14.0%)
	quite	30 (19.7%)	19 (12.8%)	42 (19.5%)	2 (8.0%)	5 (8.3%)	34 (15.2%)	15 (19.7%)	49 (16.3%)
	moderately	38 (25.0%)	41 (27.7%)	55 (25.6%)	5 (20.0%)	19 (31.7%)	59 (26.3%)	20 (26.3%)	79 (26.3%)
	slightly	23 (15.1%)	40 (27.0%)	44 (20.5%)	10 (40.0%)	9 (15.0%)	44 (19.6%)	19 (25.0%)	63 (21.0%)
	not at all	32 (21.1%)	31 (20.9%)	39 (18.1%)	6 (24.0%)	18 (30.0%)	50 (22.3%)	13 (17.1%)	63 (21.0%)
	missing	2 (1.3%)	2 (1.4%)	4 (1.9%)	0 (0%)	0 (0%)	4 (1.8%)	0 (0%)	4 (1.3%)
Seeing/Hearing	very	14 (9.2%)	14 (9.5%)	21 (9.8%)	**0 (0%)**	7 (11.7%)	20 (8.9%)	8 (10.5%)	28 (9.3%)
	quite	16 (10.5%)	21 (14.2%)	25 (11.6%)	4 (16.0%)	8 (13.3%)	27 (12.1%)	10 (13.2%)	37 (12.3%)
	moderately	25 (16.4%)	22 (14.9%)	32 (14.9%)	5 (20.0%)	10 (16.7%)	36 (16.1%)	11 (14.5%)	47 (15.7%)
	slightly	21 (13.8%)	29 (19.6%)	40 (18.6%)	3 (12.0%)	7 (11.7%)	37 (16.5%)	13 (17.1%)	50 (16.7%)
	not at all	76 (50.0%)	61 (41.2%)	96 (44.7%)	13 (52.0%)	28 (46.7%)	103 (46.0%)	34 (44.7%)	137 (45.7%)
	missing	0 (0%)	1 (0.7%)	1 (0.5%)	**0 (0%)**	0 (0%)	1 (0.4%)	0 (0%)	1 (0.3%)
TBI Effects	very	11 (7.2%)	8 (5.4%)	11 (5.1%)	0 (0%)	8 (13.3%)	15 (6.7%)	4 (5.3%)	19 (6.3%)
	quite	7 (4.6%)	8 (5.4%)	3 (1.4%)	3 (12.0%)	9 (15.0%)	10 (4.5%)	5 (6.6%)	15 (5.0%)
	moderately	26 (17.1%)	10 (6.8%)	24 (11.2%)	3 (12.0%)	9 (15.0%)	24 (10.7%)	12 (15.8%)	36 (12.0%)
	slightly	23 (15.1%)	34 (23.0%)	39 (18.1%)	5 (20.0%)	13 (21.7%)	39 (17.4%)	18 (23.7%)	57 (19.0%)
	not at all	85 (55.9%)	88 (59.5%)	138 (64.2%)	14 (56.0%)	21 (35.0%)	136 (60.7%)	37 (48.7%)	173 (57.7%)
	missing	0 (0%)	0 (0%)	0 (0%)	0 (0%)	0 (0%)	0 (0%)	0 (0%)	0 (0%)

Note. * Negatively worded scale measuring being bothered by TBI consequences. Study setting: face-to-face interviews conducted either offline or online (via video call). **Bold** values indicate response categories with zero endorsements.

## Appendix C. Threshold Parameters

**Table A8:** Characteristics of the threshold parameters.

		Location	T1	T2	T3	T4	D1	D2	D3	No.
Cognition	Concentration	0.66	0.01	−1.24	0.97	2.90	1	0	0	1
	Talking to Others	−0.04	−0.15	−0.89	−0.06	0.94	1	0	0	1
	Remembering	0.57	−0.18	−0.54	1.13	1.86	1	0	0	1
	Thinking Speed	0.32	−1.17	−0.19	0.47	2.16	0	0	0	**0**
	Planning	0.53	0.34	−0.64	0.90	1.54	1	0	0	1
	Orientation	−0.84	−2.19	−0.02	−0.31	n.a.	0	1	n.a.	1
	Decision Between Two	1.12	0.20	−0.31	1.39	3.19	1	0	0	1
Self	Appearance	0.83	−0.89	−0.64	1.57	3.27	0	0	0	**0**
	Self-Esteem	0.69	−0.38	−0.69	1.26	2.58	1	0	0	1
	Accomplishment	−0.13	−1.31	−0.84	1.75	n.a.	0	0	n.a.	**0**
	Future	0.57	−1.14	0.05	0.89	2.49	0	0	0	**0**
	Energy	0.91	−1.35	0.13	1.70	3.14	0	0	0	**0**
Autonomy & Daily Life	Manage at School	0.65	−0.54	−0.03	0.61	2.55	0	0	0	**0**
	Decision Making	0.43	0.74	−1.22	0.30	1.92	1	0	0	1
	Daily Independence	−0.30	−0.23	−0.29	−0.18	−0.48	1	0	1	2
	Getting out and About	0.25	−0.12	−0.07	0.55	0.64	0	0	0	**0**
	Social Activities	0.51	0.96	−0.21	0.03	1.25	1	0	0	1
	Support from Others	0.06	−0.05	−1.35	−0.13	1.77	1	0	0	1
	Ability to Move	−0.14	−1.18	0.25	0.04	0.34	0	1	0	1
Social	Family Relationship	0.71	0.51	−1.09	0.83	2.59	1	0	0	1
	Relationship with Friends	0.05	−0.51	−0.45	−0.26	1.44	0	0	0	**0**
	Attitudes of Others	0.46	−1.66	0.21	0.64	2.63	0	0	0	**0**
	Friendships	0.03	−1.02	0.07	−0.06	1.12	0	1	0	1
	Open up to Others	0.65	−1.09	0.46	0.61	2.62	0	0	0	**0**
	Demands from Others	1.00	−0.21	−0.36	0.92	3.63	1	0	0	1
Emotions	Anger	0.42	−0.64	−0.19	0.95	1.58	0	0	0	**0**
	Anxiety	0.19	−0.82	−0.30	1.02	0.87	0	0	1	1
	Sadness	0.41	−0.53	0.24	0.88	1.05	0	0	0	**0**
	Loneliness	0.05	−0.22	−0.06	0.14	0.36	0	0	0	**0**
Physical	TBI Effects	−0.40	−0.15	−0.84	−0.11	−0.49	1	0	1	2
	Headaches	0.46	0.04	0.42	0.60	0.77	0	0	0	**0**
	Pain	0.37	−0.04	−0.13	0.80	0.84	1	0	0	1
	Clumsiness	0.44	−0.36	−0.19	0.98	1.33	0	0	0	**0**
	Seeing/Hearing	−0.08	−0.31	−0.07	0.32	−0.24	0	0	1	1
	Other Injuries	−0.20	−0.20	−0.40	0.60	−0.81	1	0	1	2

Note. Location = item location parameter, T1–T4 = response categories’, D1–D3 = logical test of whether the threshold value in category k is lower than in category k + 1 (0 = no disorder, 1 = disorder), No. = number of disordered thresholds per item (sum from D1 to D3), n.a. = not available (due to missing responses). **Bold** values indicate absence of threshold disorder.
