# Supplementary material for: Psychometric Properties of the German Version of the Quality of Life after Brain Injury Scale for Kids and Adolescents (QOLIBRI-KID/ADO) Using Item Response Theory Framework: Results from the Pilot Study
Source: J Clin Med. 2023 May 27;12(11):3716. doi: 10.3390/jcm12113716 (PMC10253612; doi:10.3390/jcm12113716)
Supplement: Supplementary file 1 [file jcm-12-03716-s001.zip › jcm-2257233-supplementary.pdf]

A: Cognition

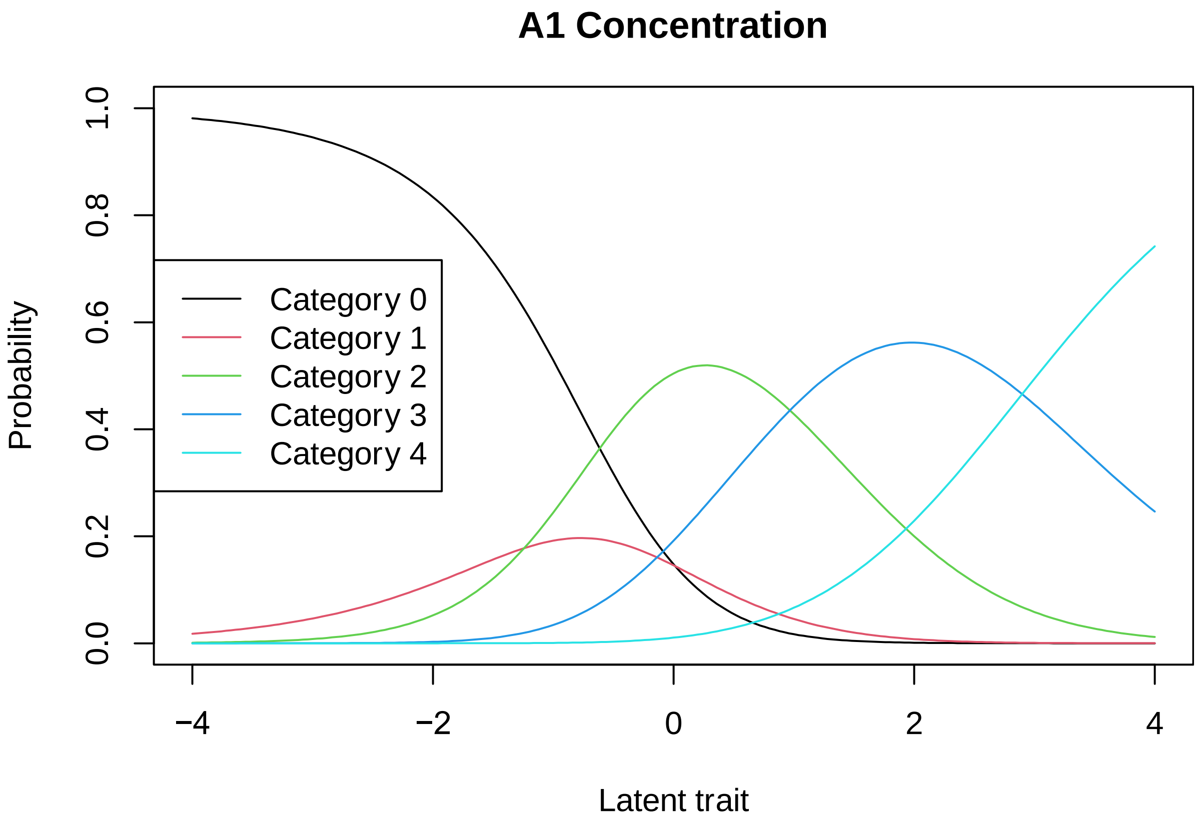
A1 Concentration
Probability
0.0
0.2
0.4
0.6
0.8
1.0
−4
−2
0
2
4
Latent trait
Category 0
Category 1
Category 2
Category 3
Category 4

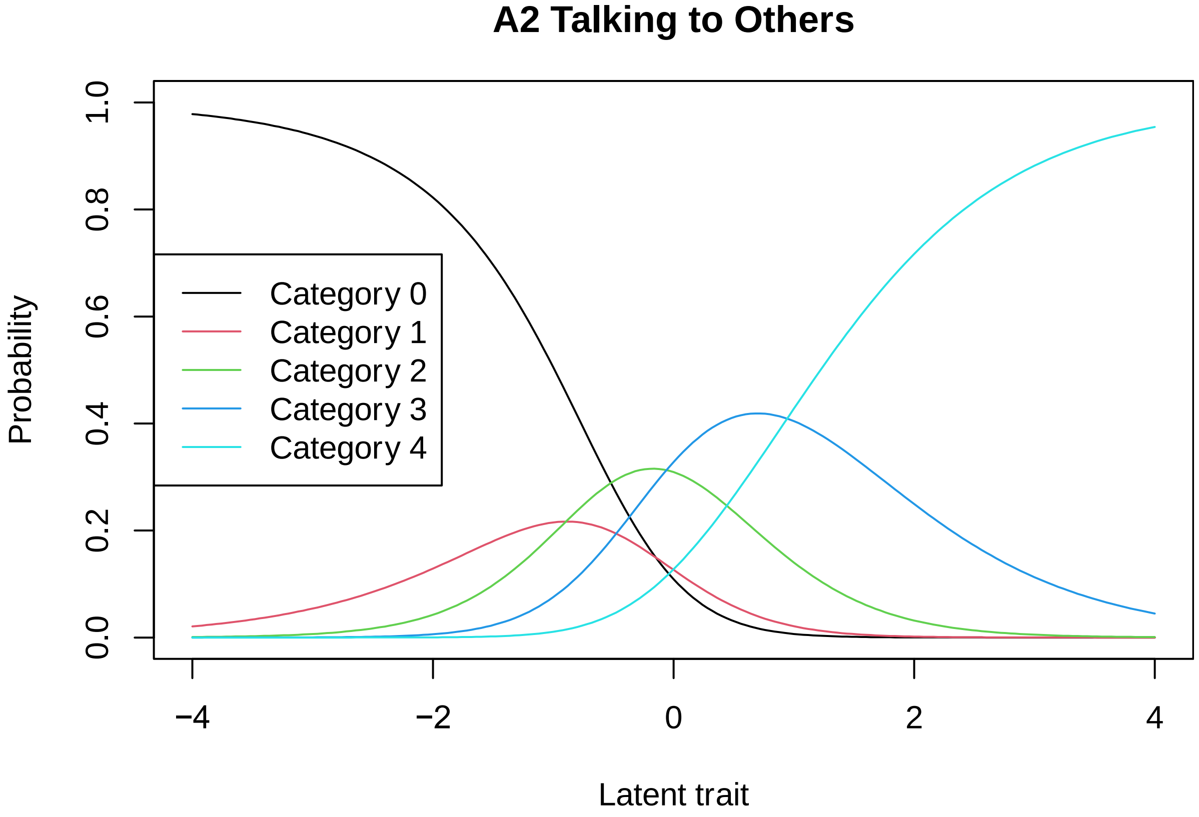
A2 Talking to Others
Probability
0.0
0.2
0.4
0.6
0.8
1.0
−4
−2
0
2
4
Latent trait
Category 0
Category 1
Category 2
Category 3
Category 4

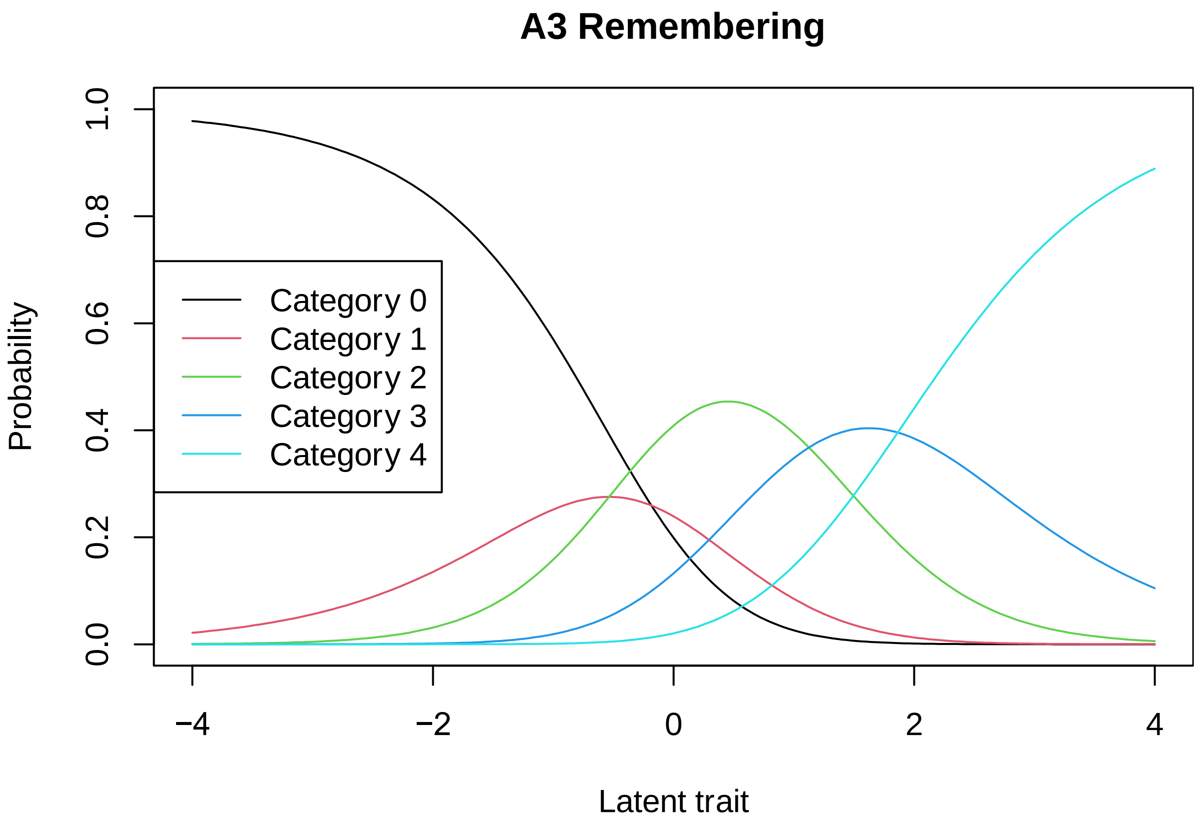
A3 Remembering
Probability
0.0
0.2
0.4
0.6
0.8
1.0
Category 0
Category 1
Category 2
Category 3
Category 4
−4
−2
0
2
4
Latent trait

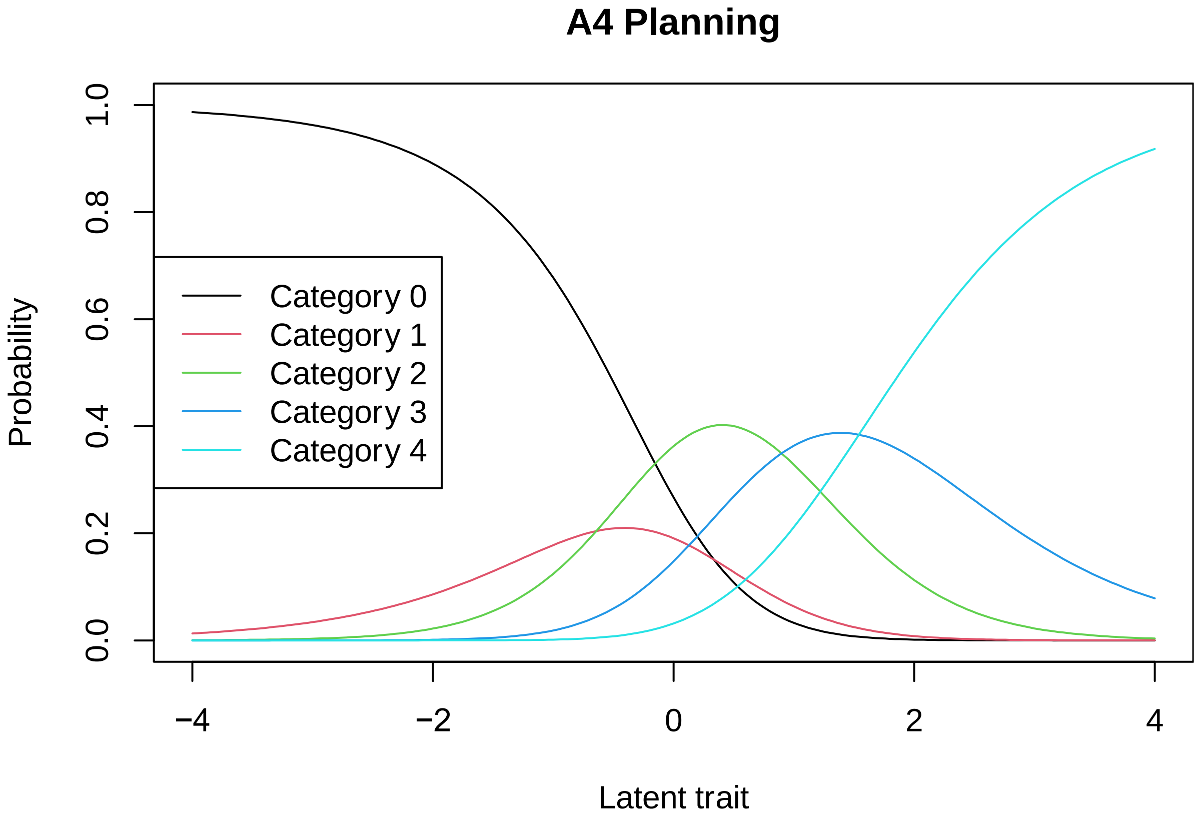
A4 Planning
Probability
0.0
0.2
0.4
0.6
0.8
1.0
Category 0
Category 1
Category 2
Category 3
Category 4
−4
−2
0
2
4
Latent trait

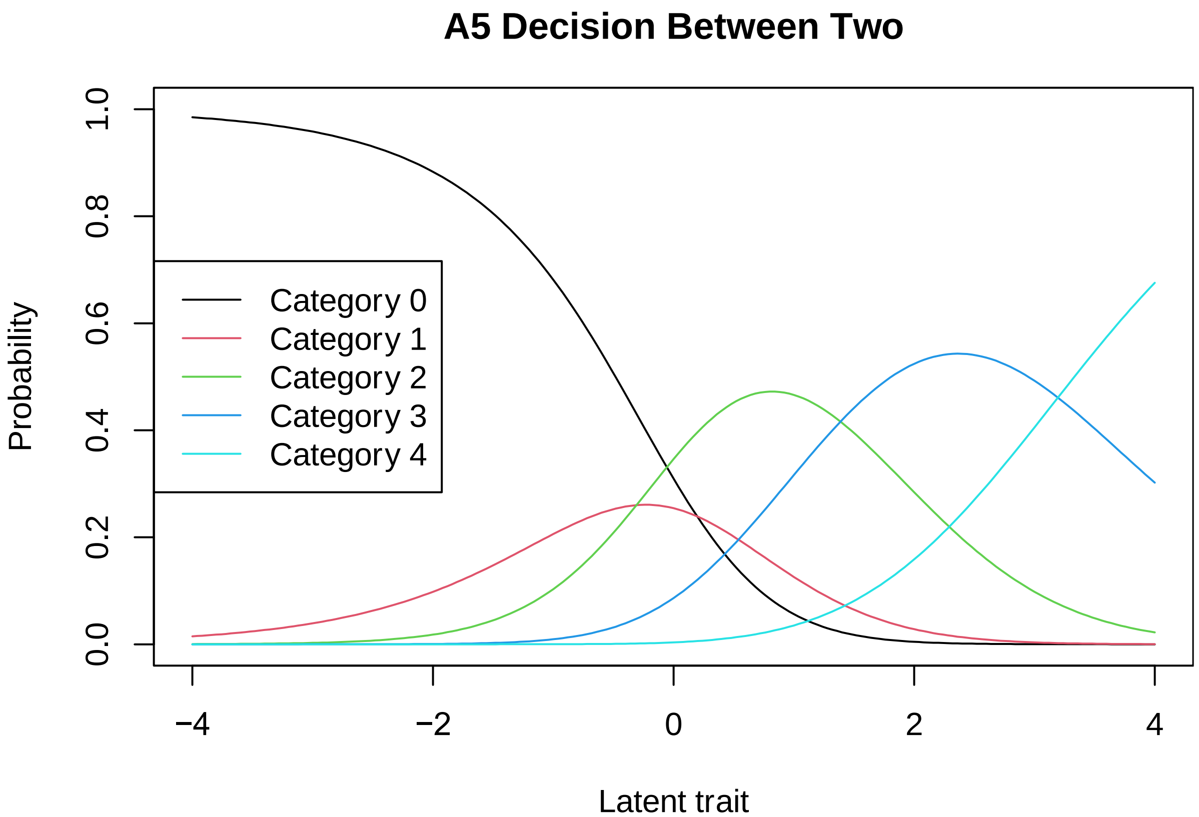
A5 Decision Between Two
Probability
1.0
0.8
0.6
0.4
0.2
0.0
Category 0
Category 1
Category 2
Category 3
Category 4
−4
−2
0
2
4
Latent trait

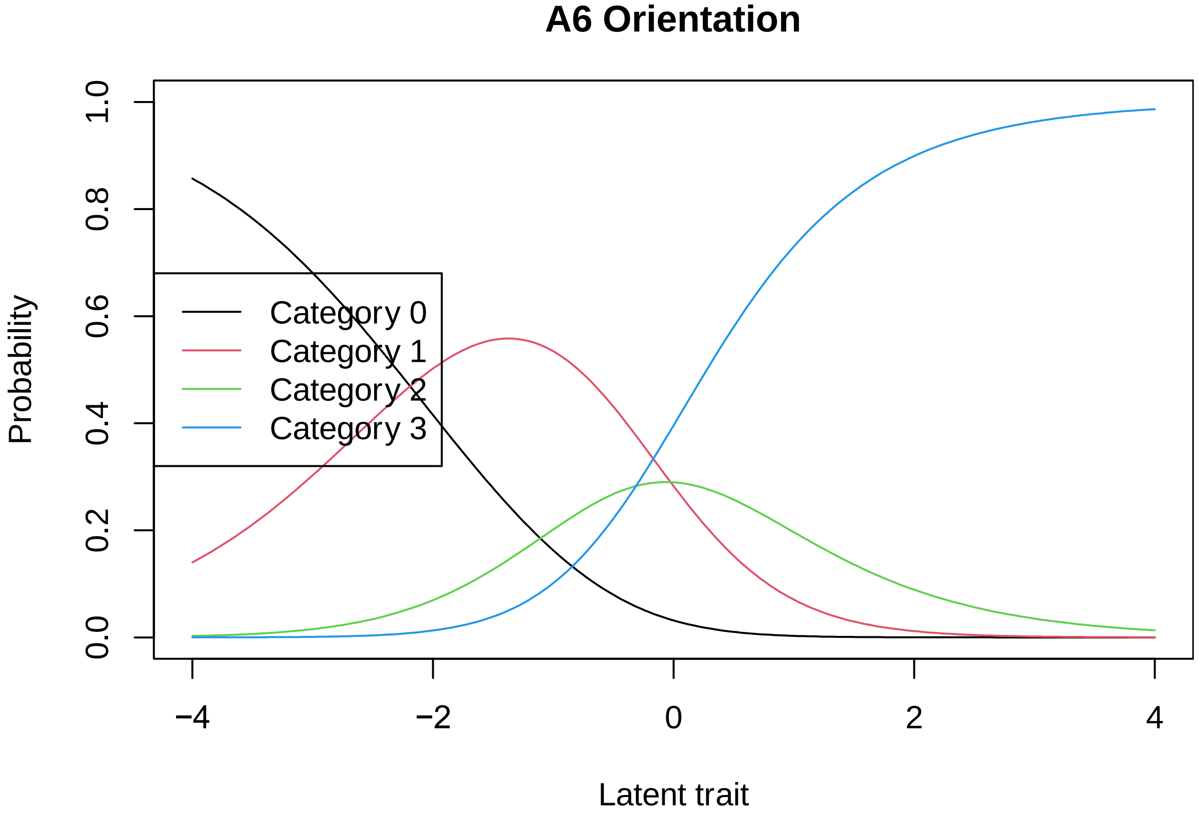
A6 Orientation
Probability
1.0
0.8
0.6
0.4
0.2
0.0
Category 0
Category 1
Category 2
Category 3
−4
−2
0
2
4
Latent trait

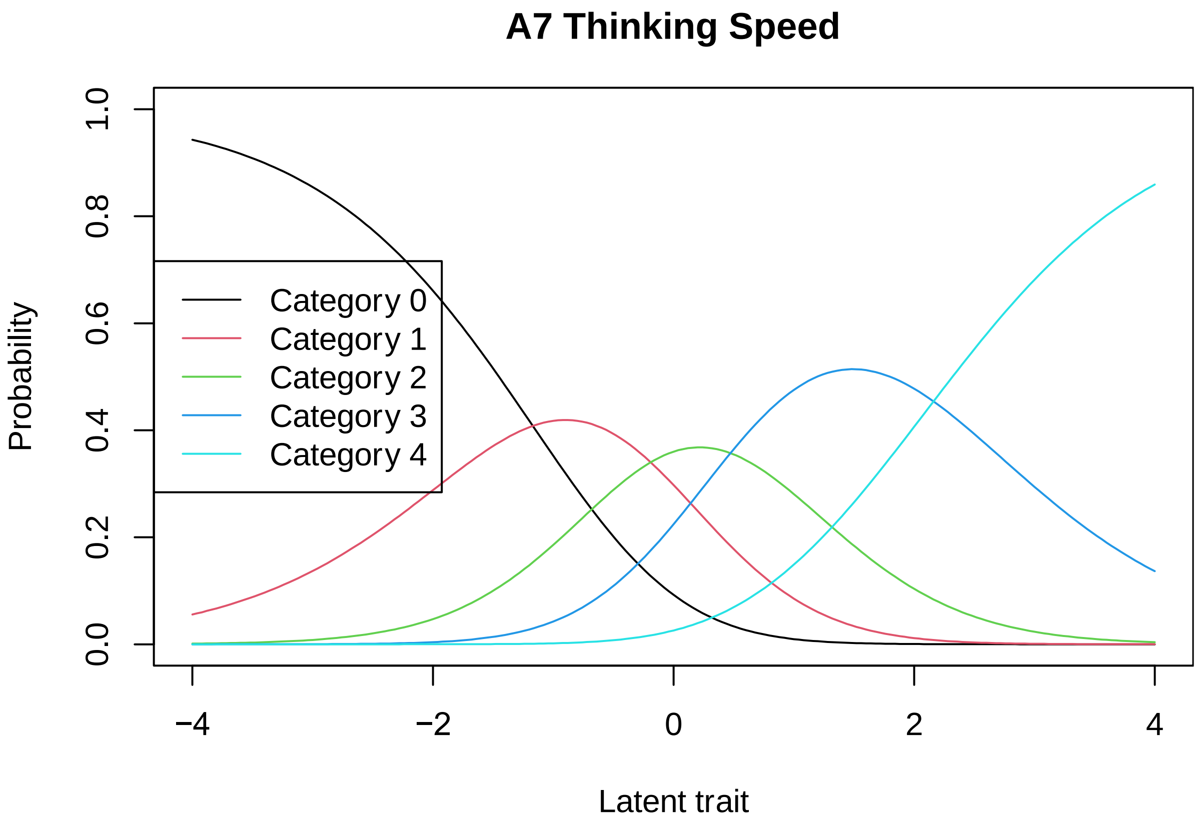
A7 Thinking Speed
Probability
1.0
0.8
0.6
0.4
0.2
0.0
Category 0
Category 1
Category 2
Category 3
Category 4
−4
−2
0
2
4
Latent trait

B: Self

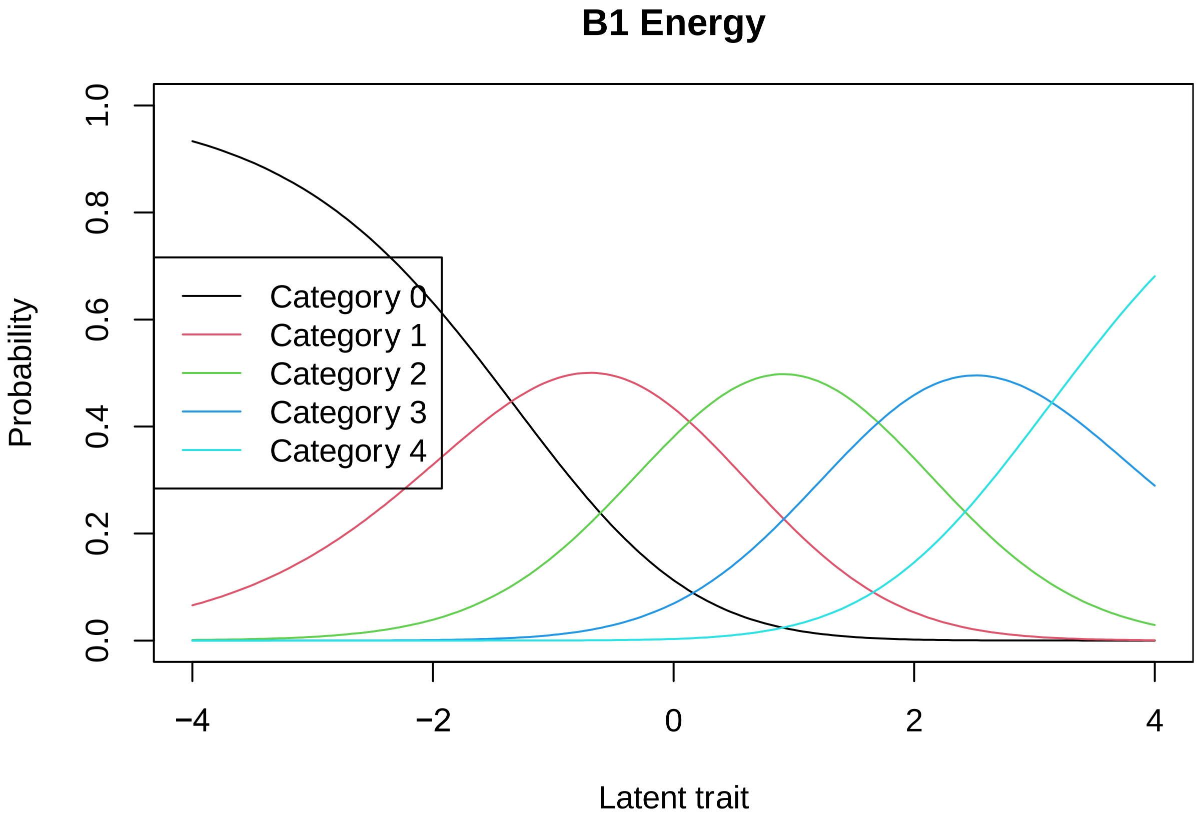
B1 Energy
Probability
Latent trait
Category 0
Category 1
Category 2
Category 3
Category 4
0.0
0.2
0.4
0.6
0.8
1.0
−4
−2
0
2
4

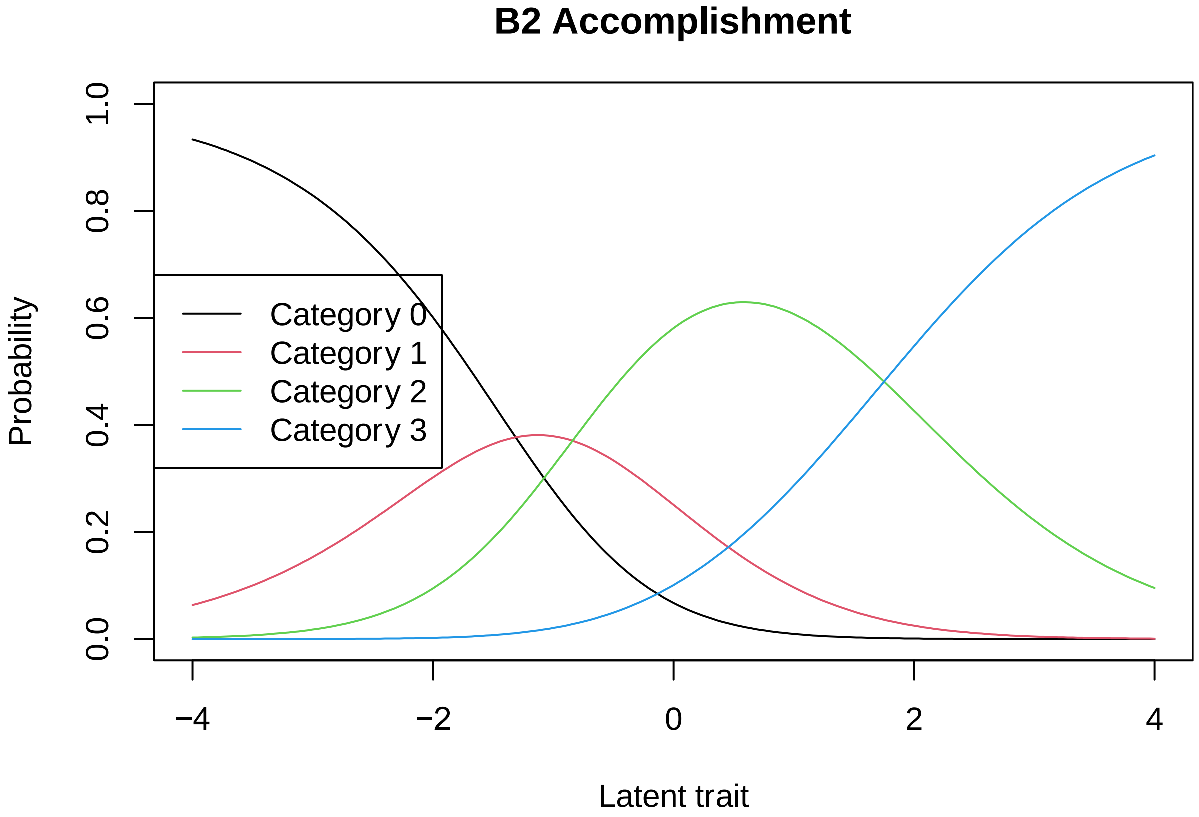
B2 Accomplishment
Probability
Latent trait
Category 0
Category 1
Category 2
Category 3
0.0
0.2
0.4
0.6
0.8
1.0
−4
−2
0
2
4

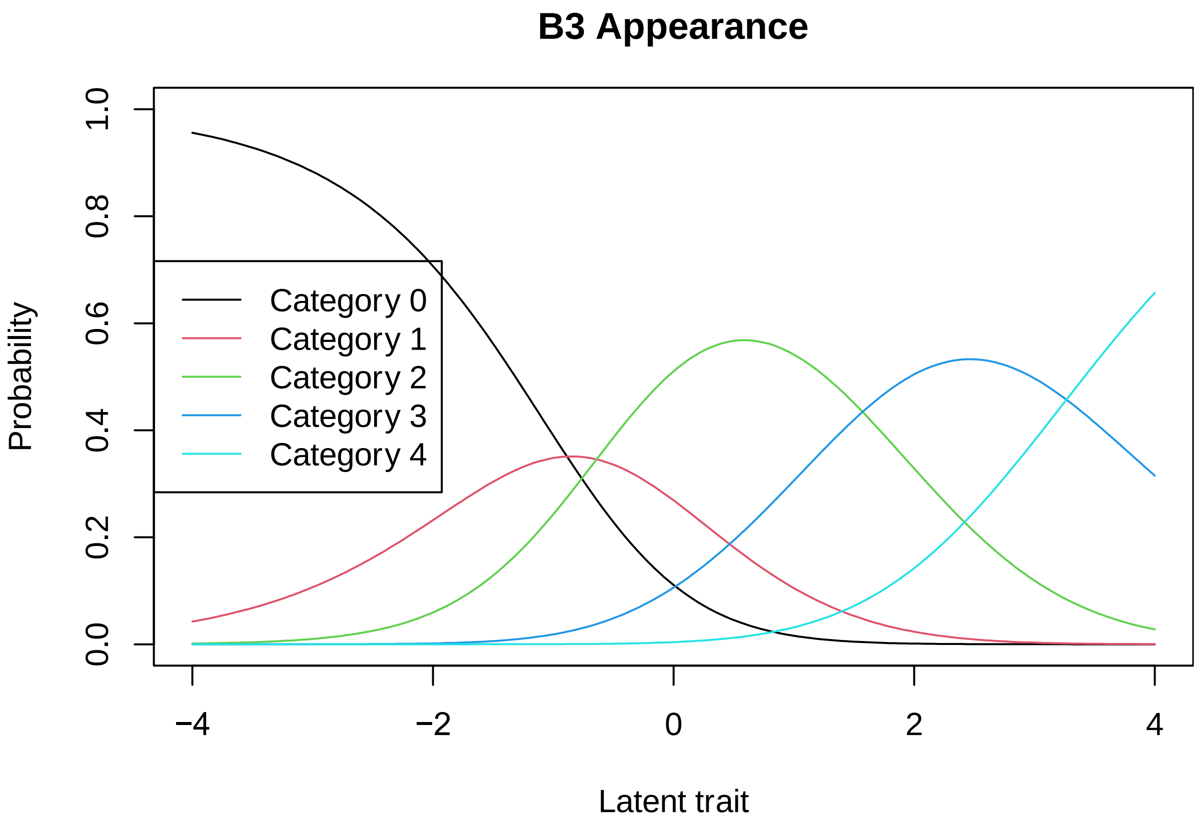
B3 Appearance
Probability
1.0
0.8
0.6
0.4
0.2
0.0
Category 0
Category 1
Category 2
Category 3
Category 4
−4
−2
0
2
4
Latent trait

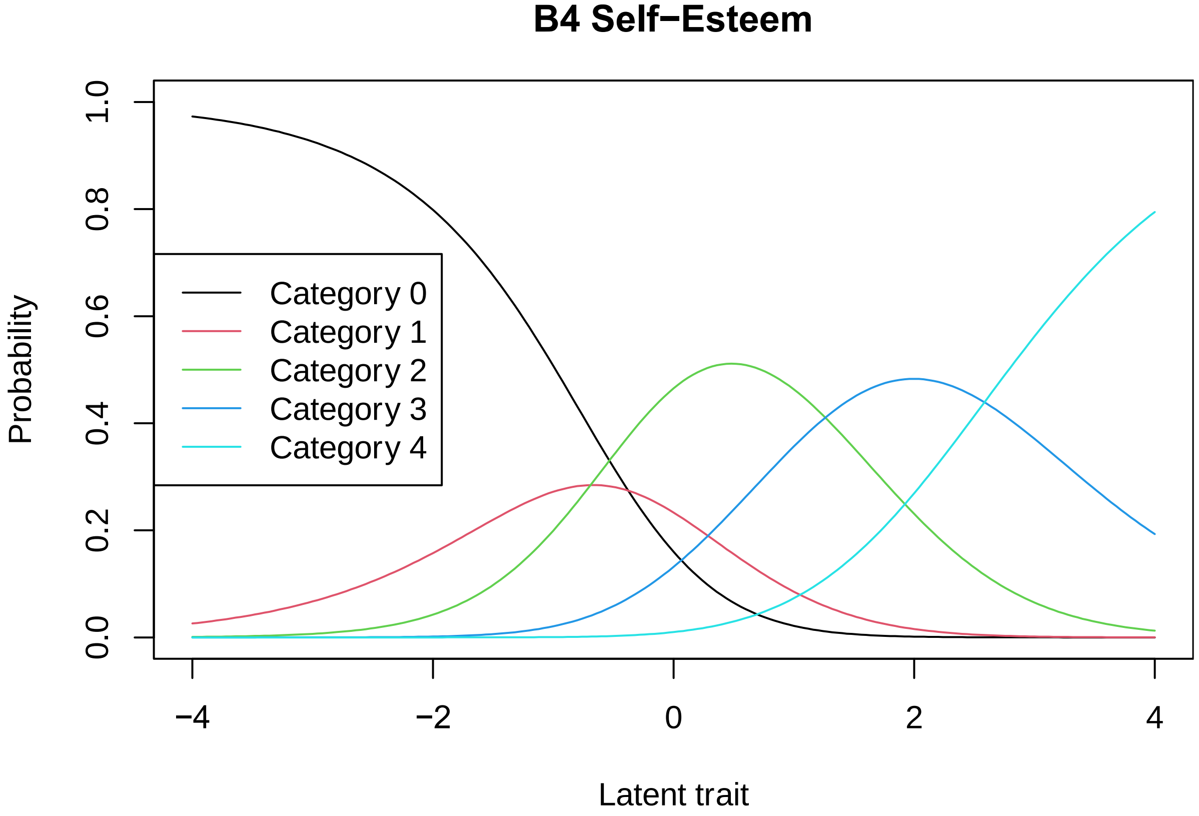
B4 Self−Esteem
Probability
1.0
0.8
0.6
0.4
0.2
0.0
Category 0
Category 1
Category 2
Category 3
Category 4
−4
−2
0
2
4
Latent trait

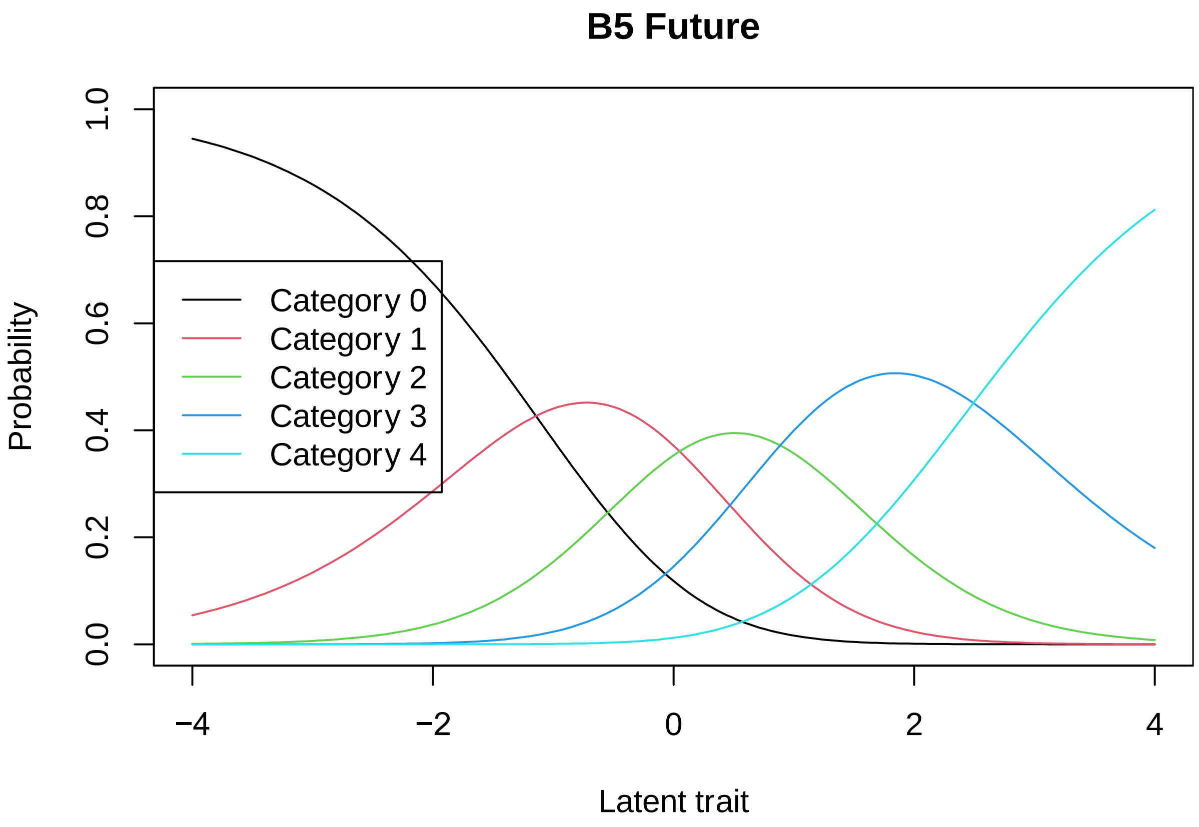
B5 Future
Probability
1.0
0.8
0.6
0.4
0.2
0.0
Category 0
Category 1
Category 2
Category 3
Category 4
−4
−2
0
2
4
Latent trait

C: Autonomy and Daily Life

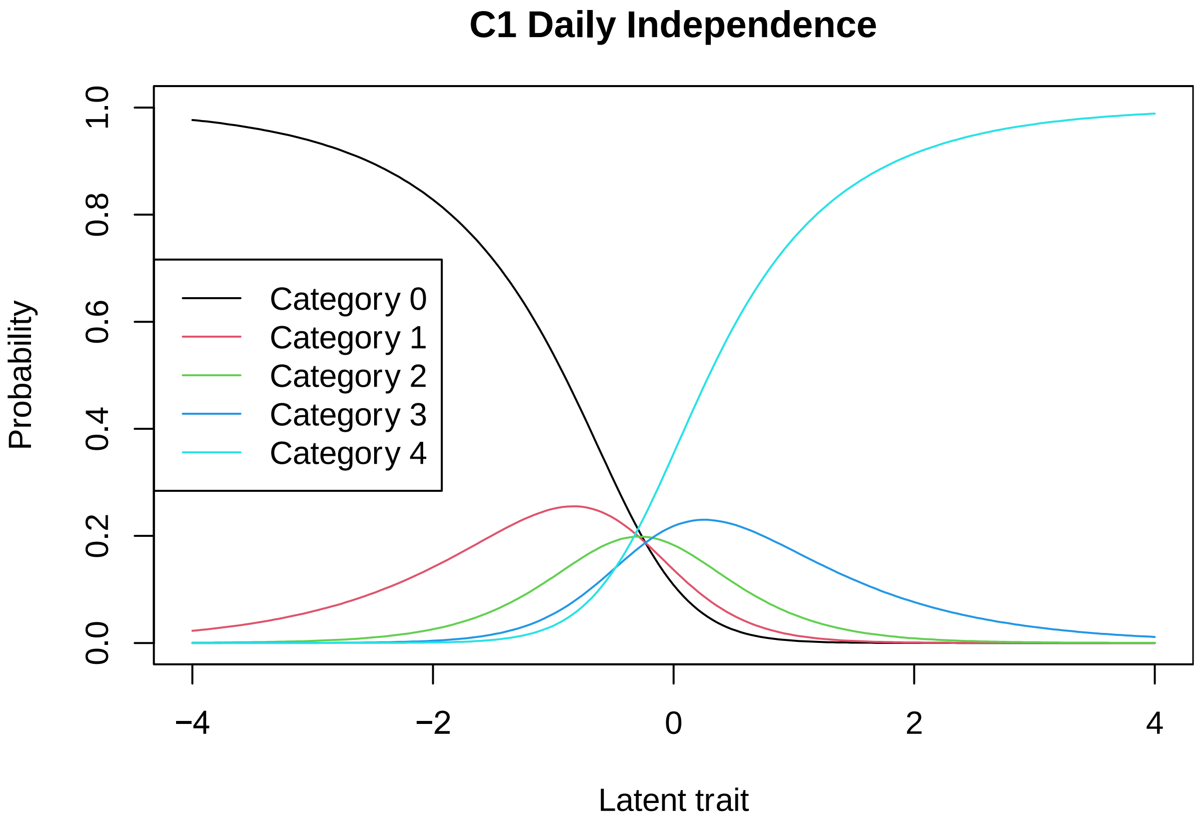

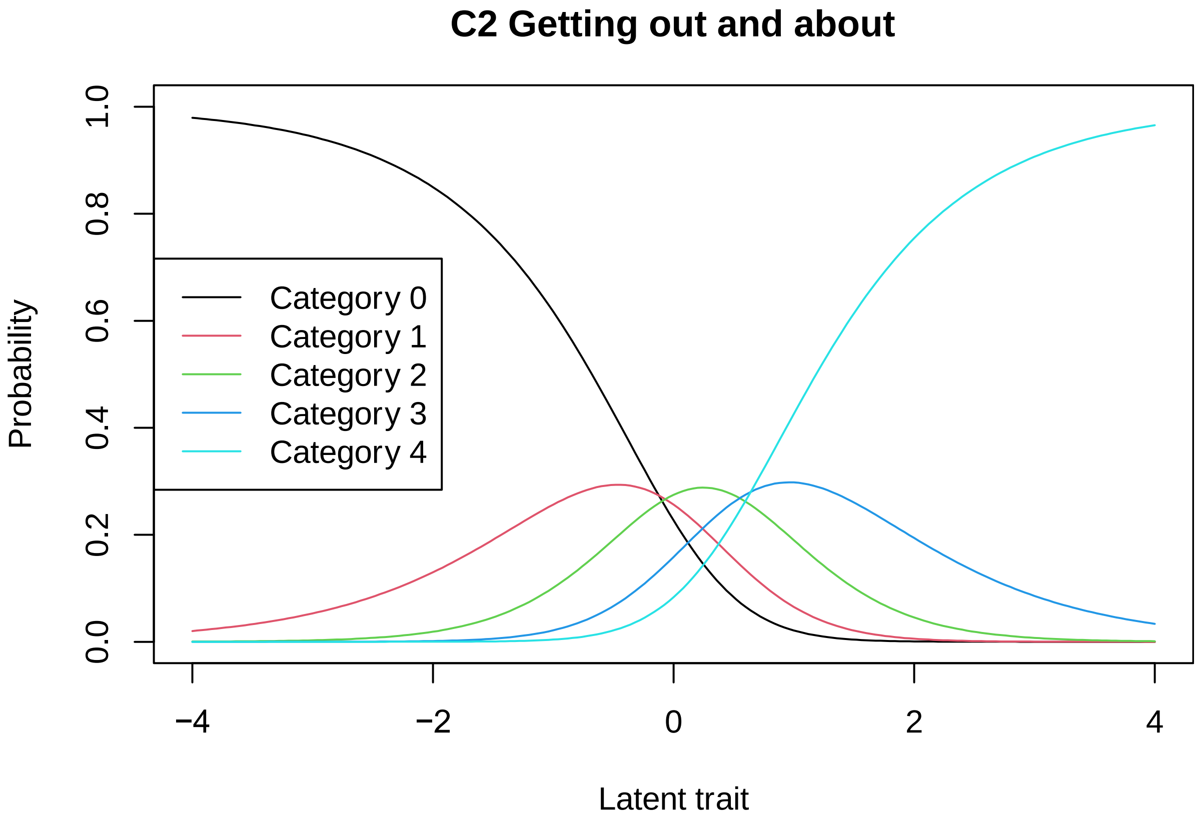

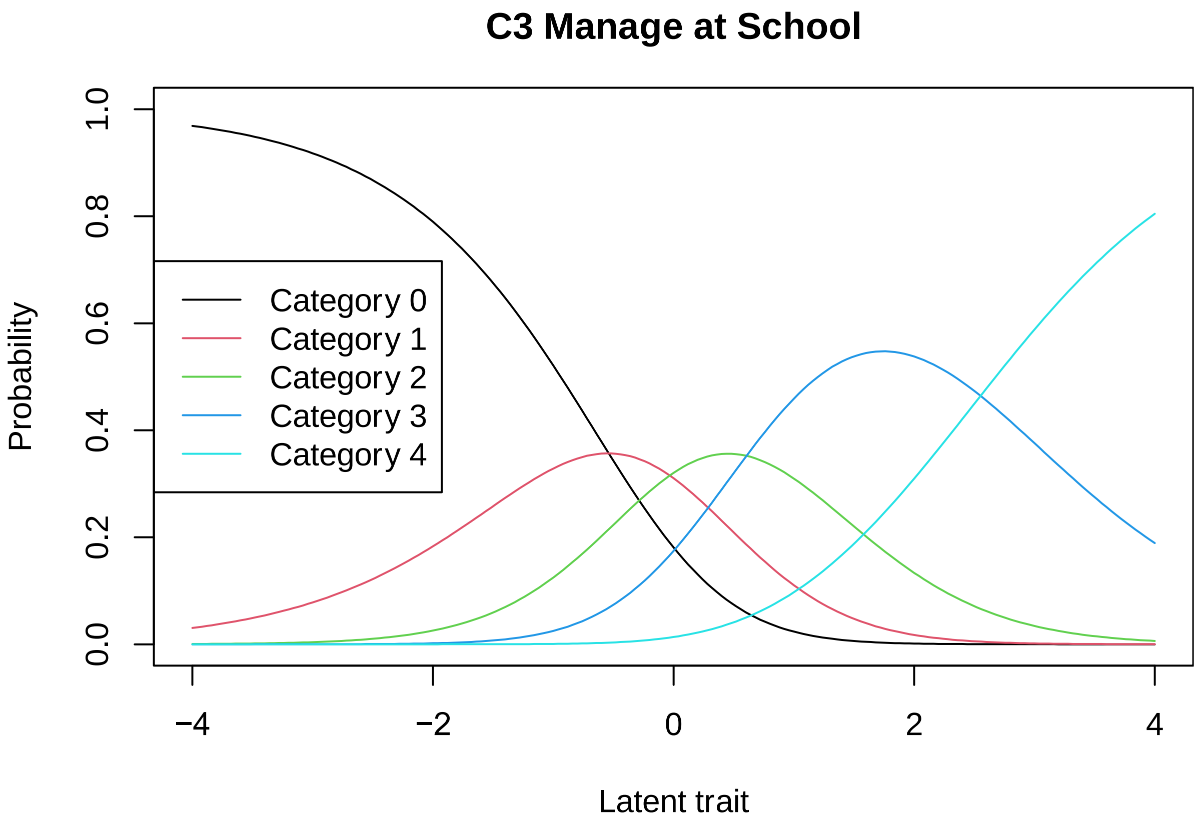
C3 Manage at School
Probability
1.0
0.8
0.6
0.4
0.2
0.0
Category 0
Category 1
Category 2
Category 3
Category 4
−4
−2
0
2
4
Latent trait

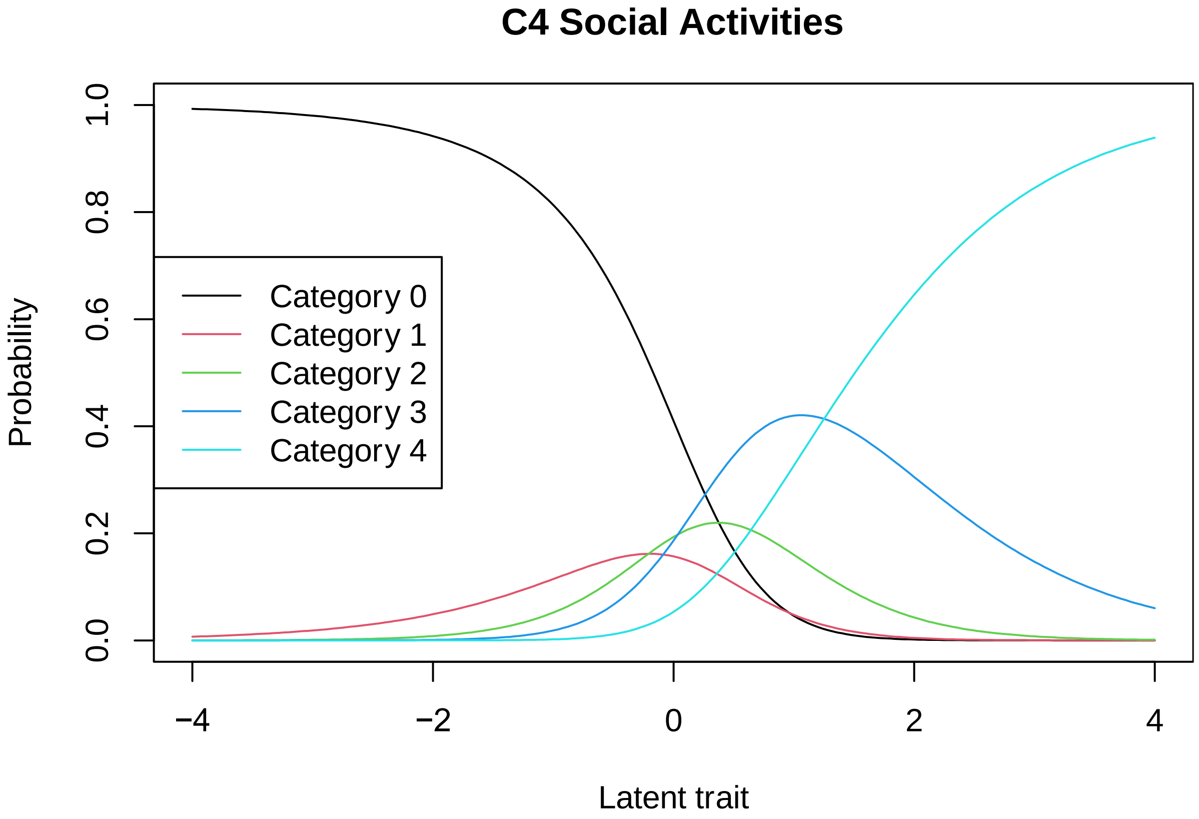
C4 Social Activities
Probability
1.0
0.8
0.6
0.4
0.2
0.0
Category 0
Category 1
Category 2
Category 3
Category 4
−4
−2
0
2
4
Latent trait

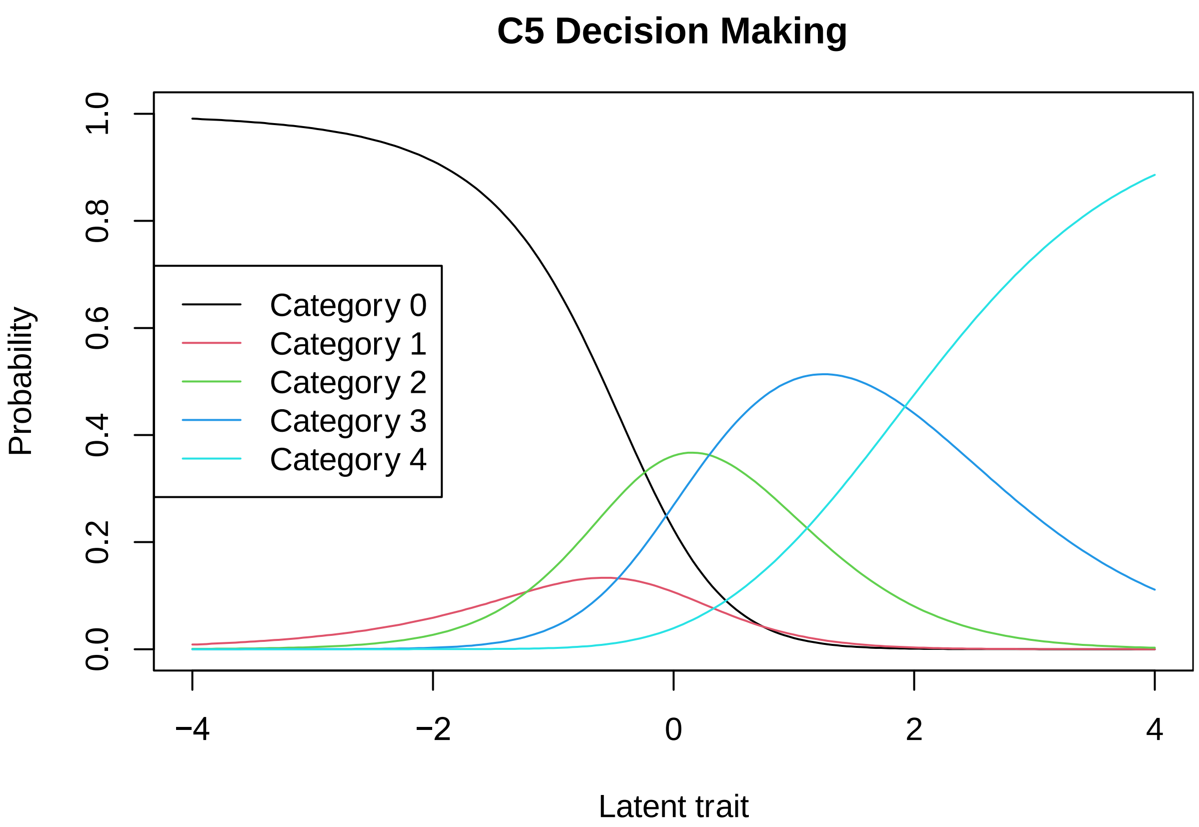
C5 Decision Making
Probability
1.0
0.8
0.6
0.4
0.2
0.0
Category 0
Category 1
Category 2
Category 3
Category 4
−4
−2
0
2
4
Latent trait

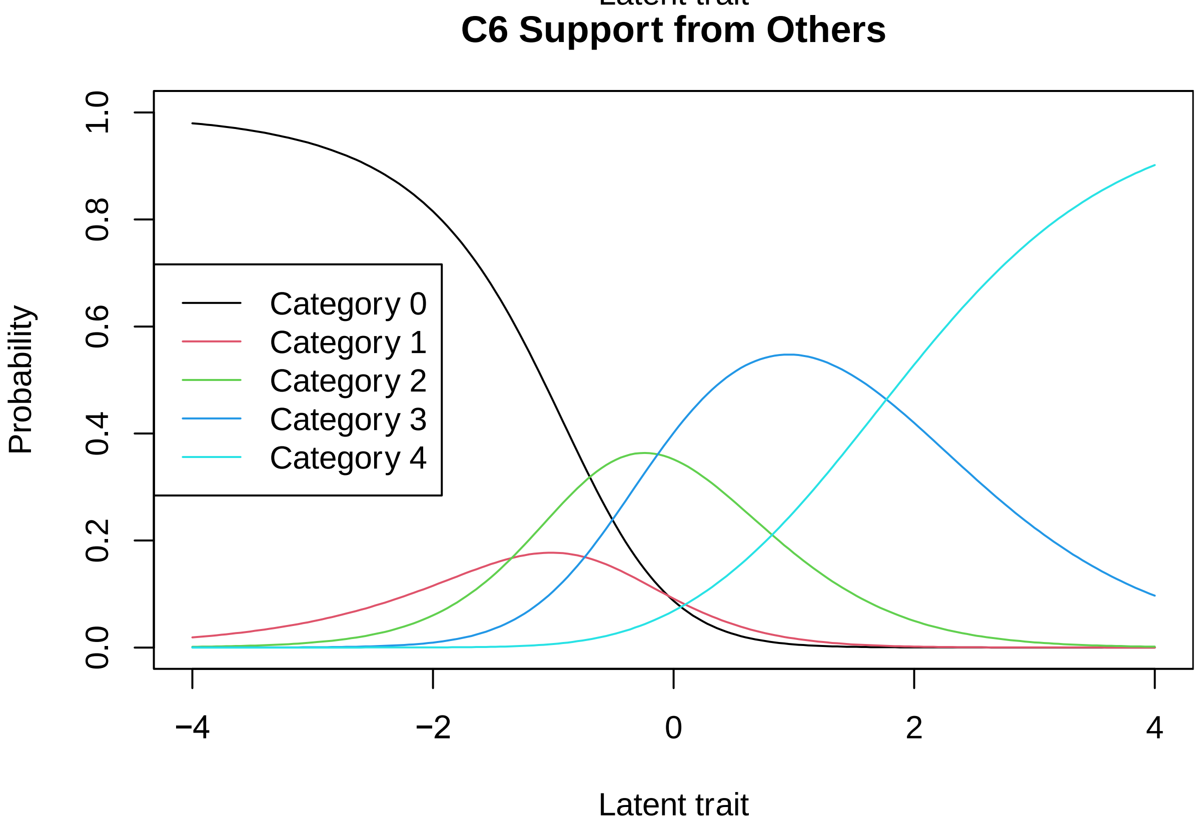
C6 Support from Others
Probability
1.0
0.8
0.6
0.4
0.2
0.0
Category 0
Category 1
Category 2
Category 3
Category 4
−4
−2
0
2
4
Latent trait

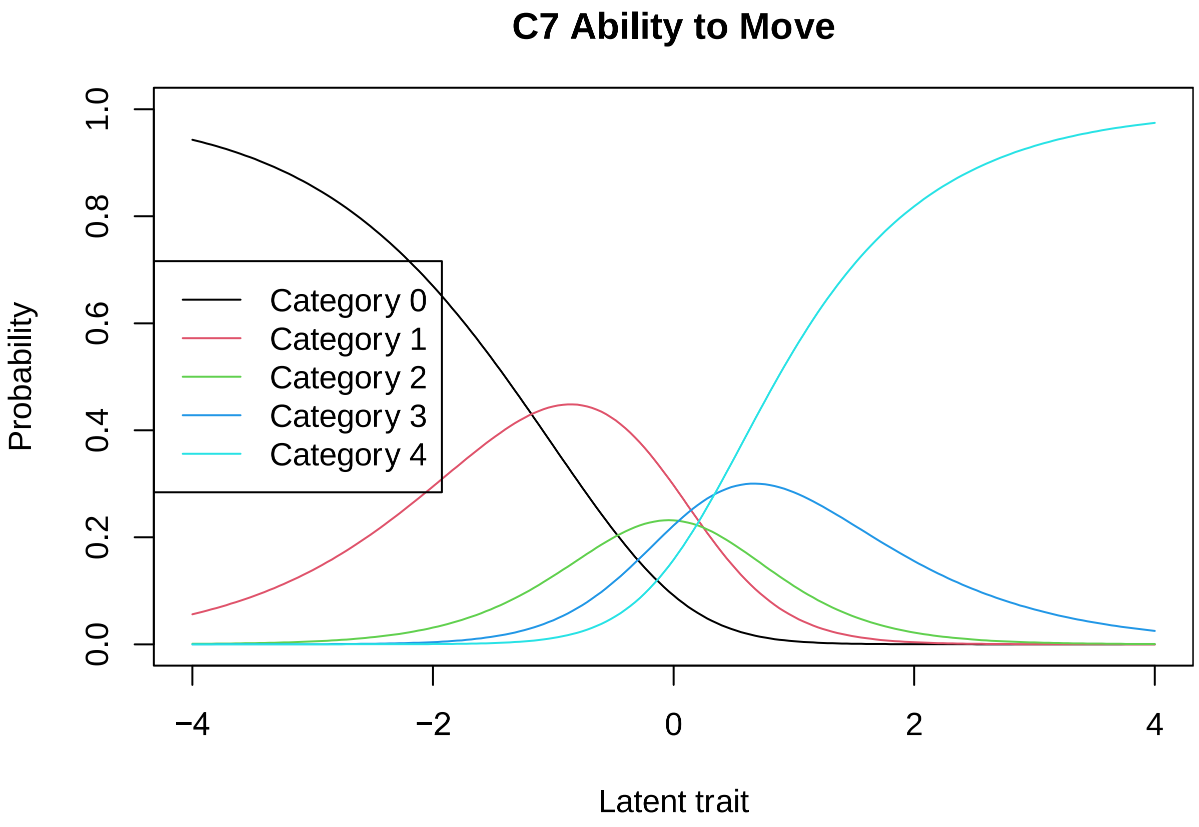
C7 Ability to Move
Probability
0.0
0.2
0.4
0.6
0.8
1.0
Category 0
Category 1
Category 2
Category 3
Category 4
−4
−2
0
2
4
Latent trait

D: Social

D1 Open up to Others

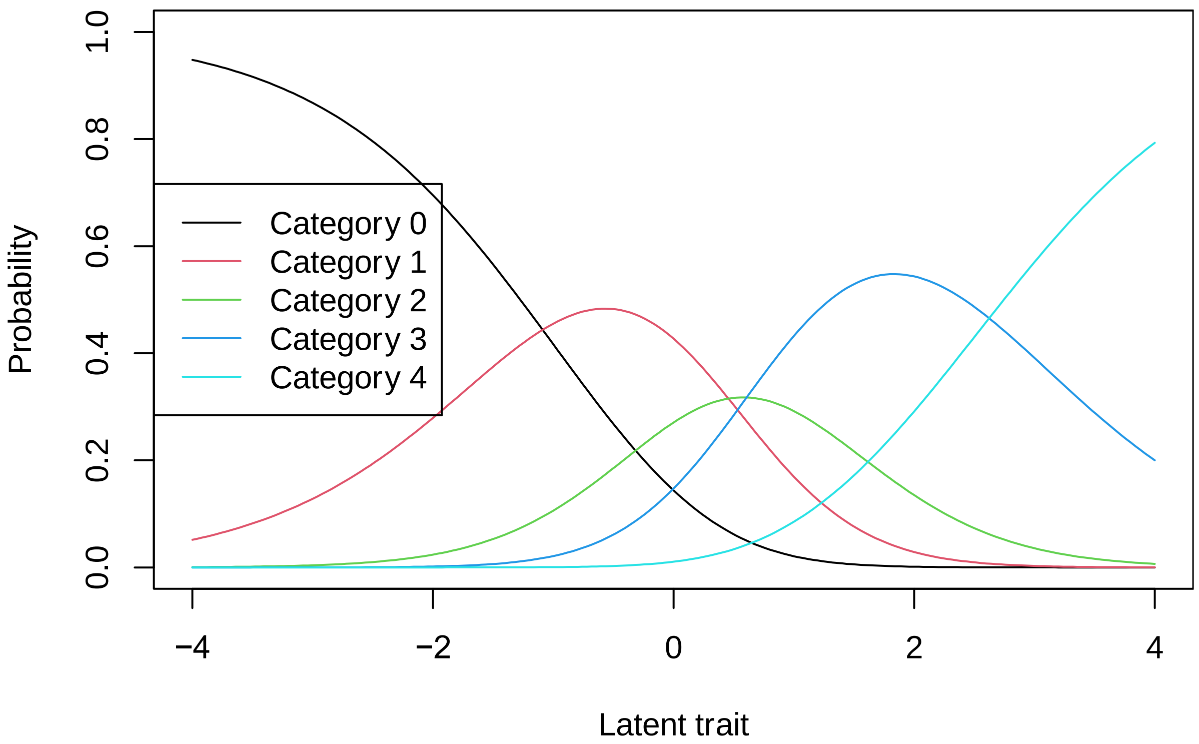

D2 Family Relationship

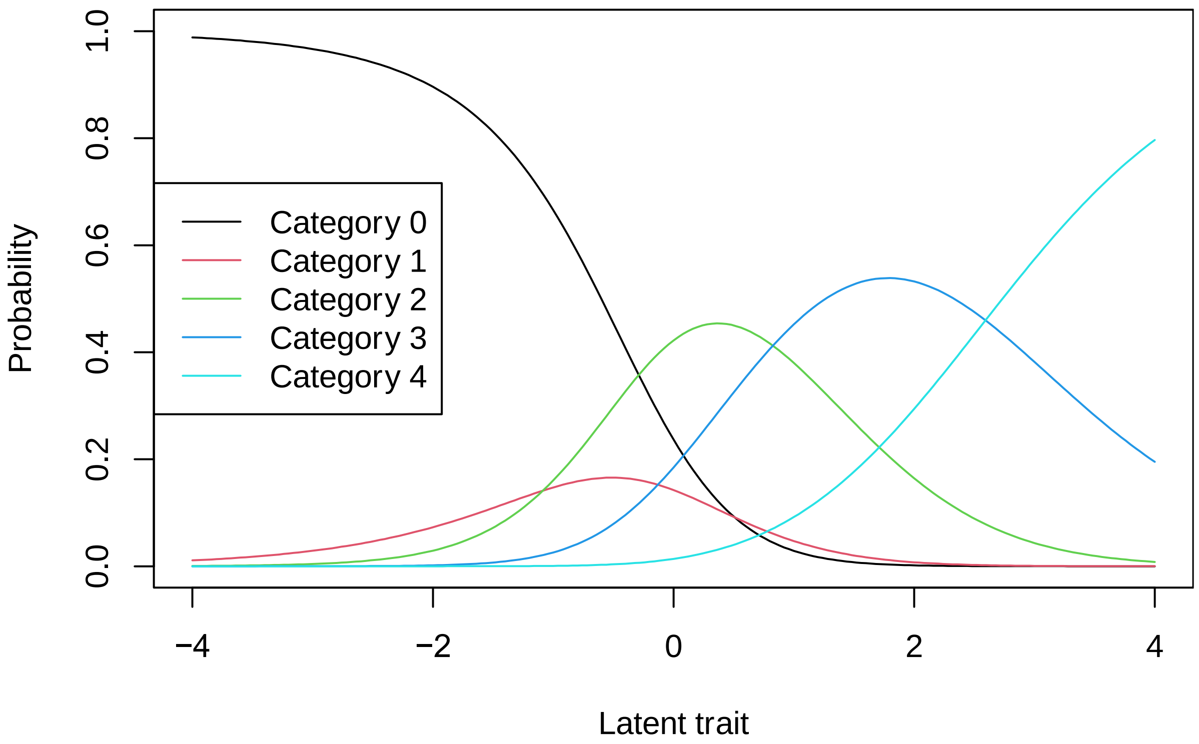

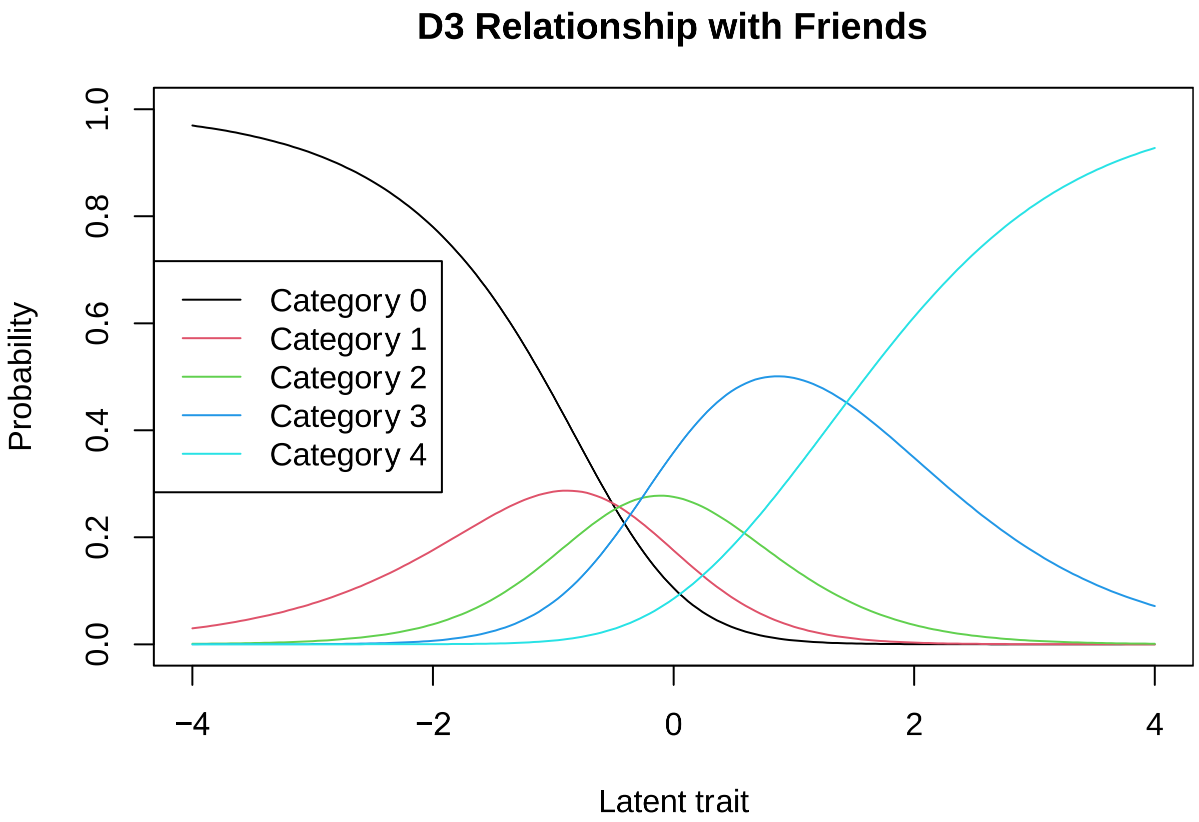
D3 Relationship with Friends
Probability
1.0
0.8
0.6
0.4
0.2
0.0
Category 0
Category 1
Category 2
Category 3
Category 4
−4
−2
0
2
4
Latent trait

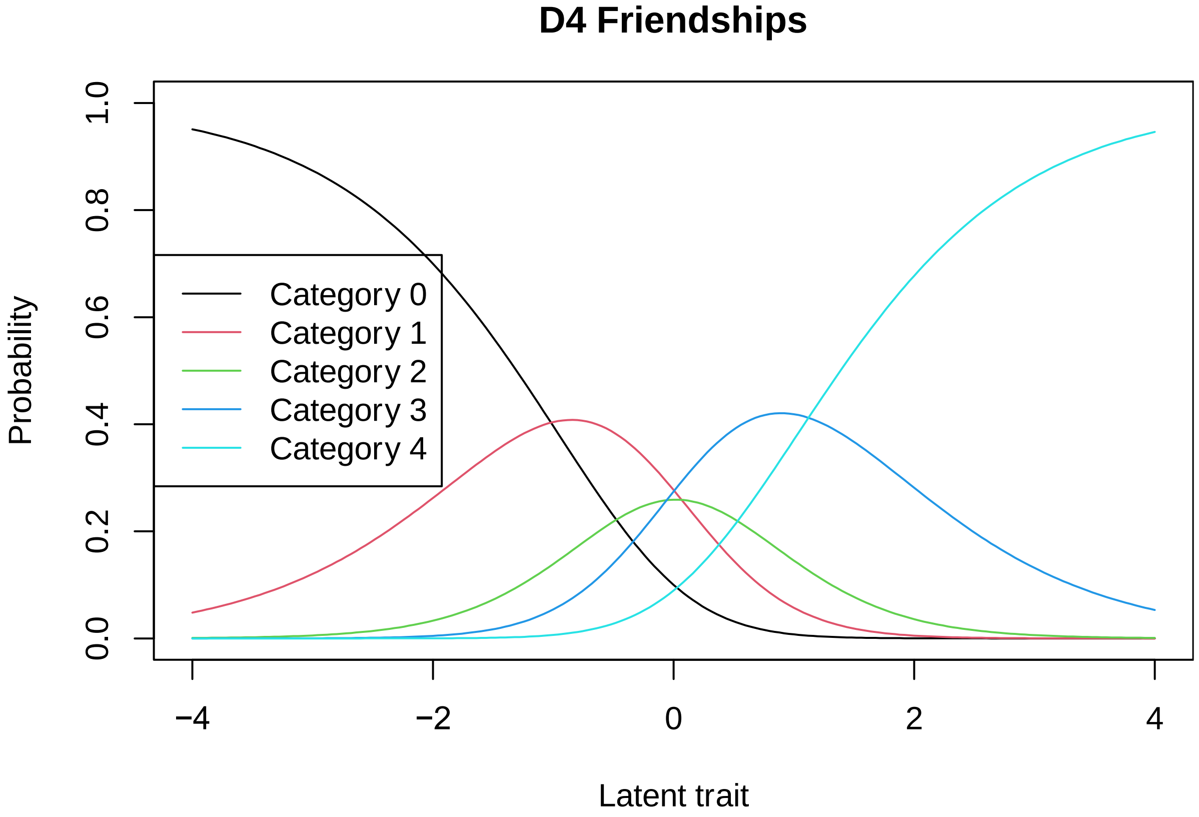
D4 Friendships
Probability
1.0
0.8
0.6
0.4
0.2
0.0
Category 0
Category 1
Category 2
Category 3
Category 4
−4
−2
0
2
4
Latent trait

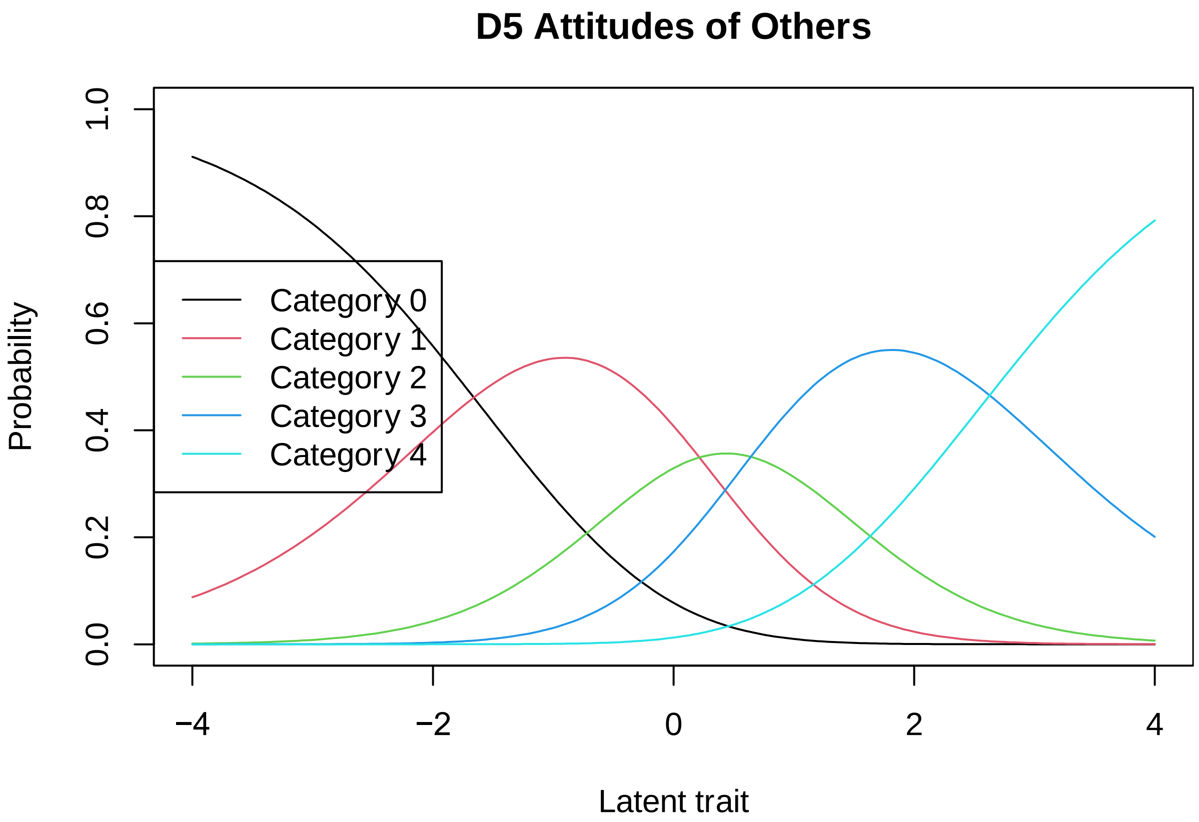
D5 Attitudes of Others
Probability
1.0
0.8
0.6
0.4
0.2
0.0
Category 0
Category 1
Category 2
Category 3
Category 4
−4
−2
0
2
4
Latent trait

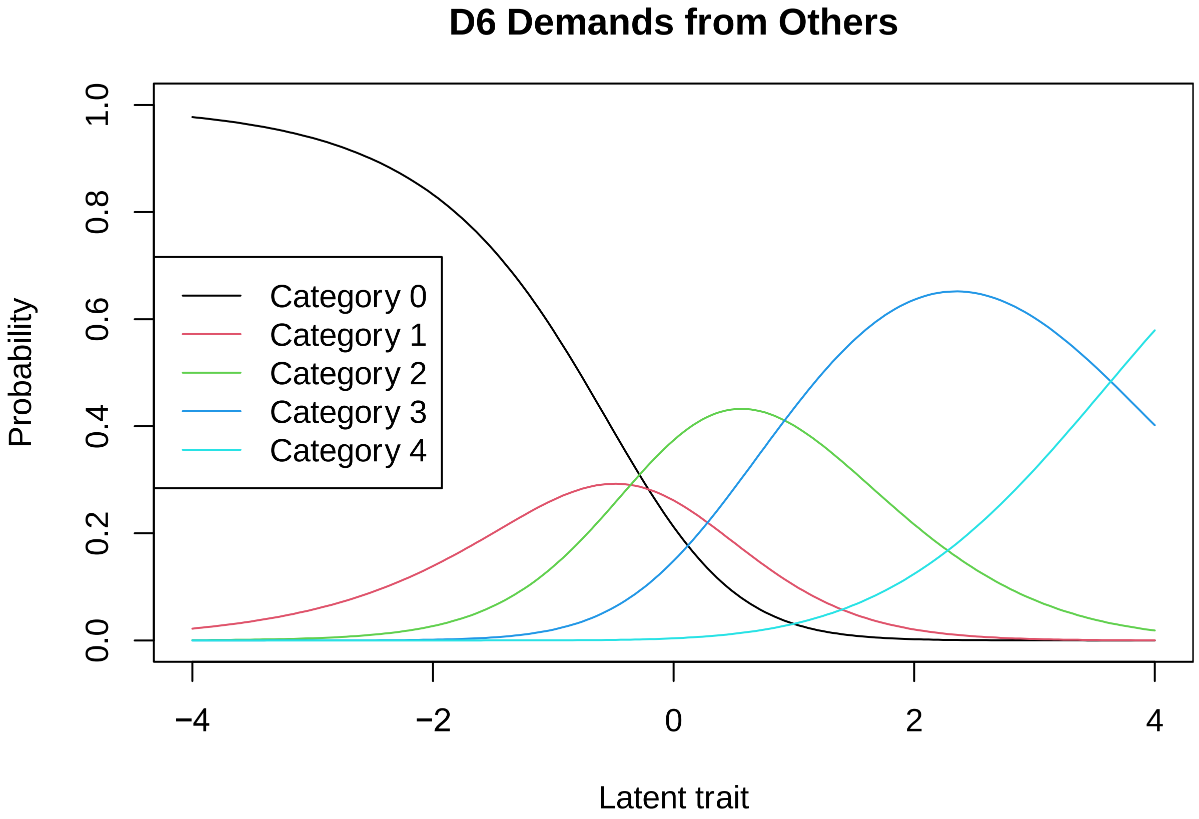
D6 Demands from Others
Probability
1.0
0.8
0.6
0.4
0.2
0.0
Category 0
Category 1
Category 2
Category 3
Category 4
−4
−2
0
2
4
Latent trait

E: Emotions

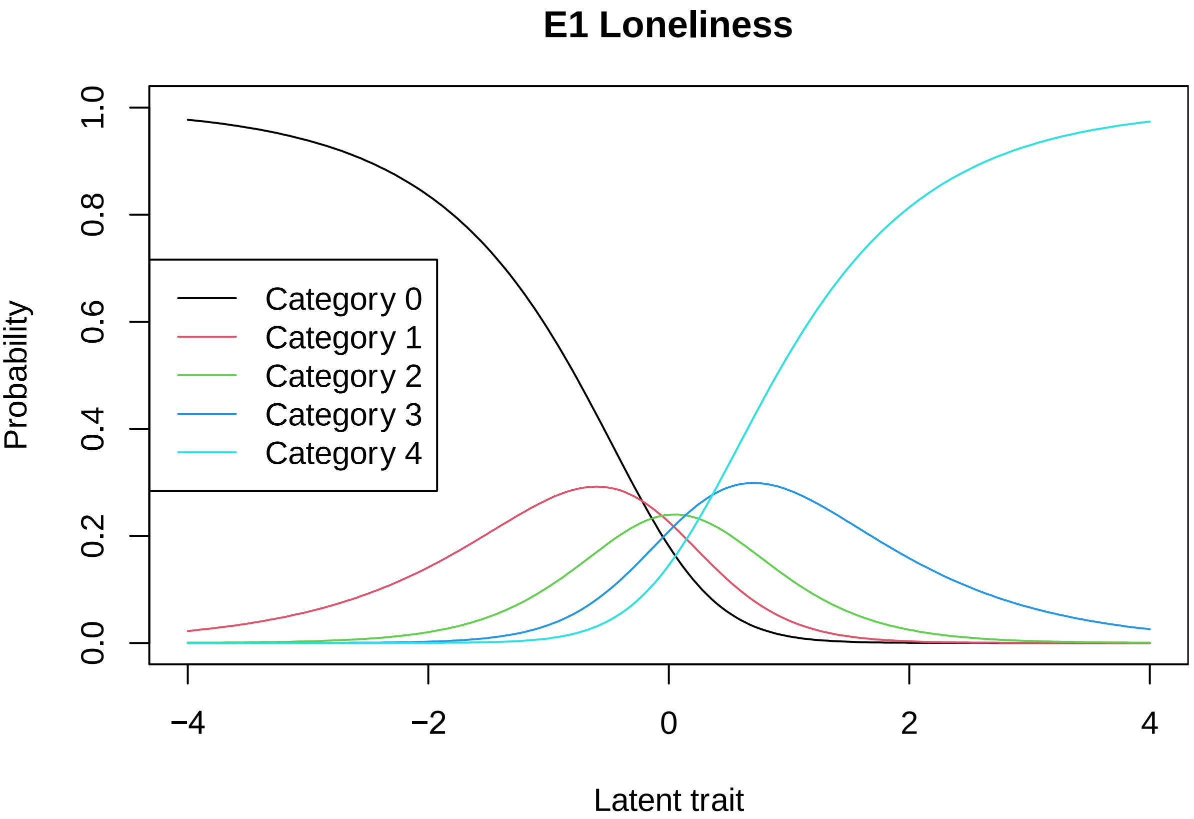
E1 Loneliness
Probability
0.0
0.2
0.4
0.6
0.8
1.0
Category 0
Category 1
Category 2
Category 3
Category 4
−4
−2
0
2
4
Latent trait

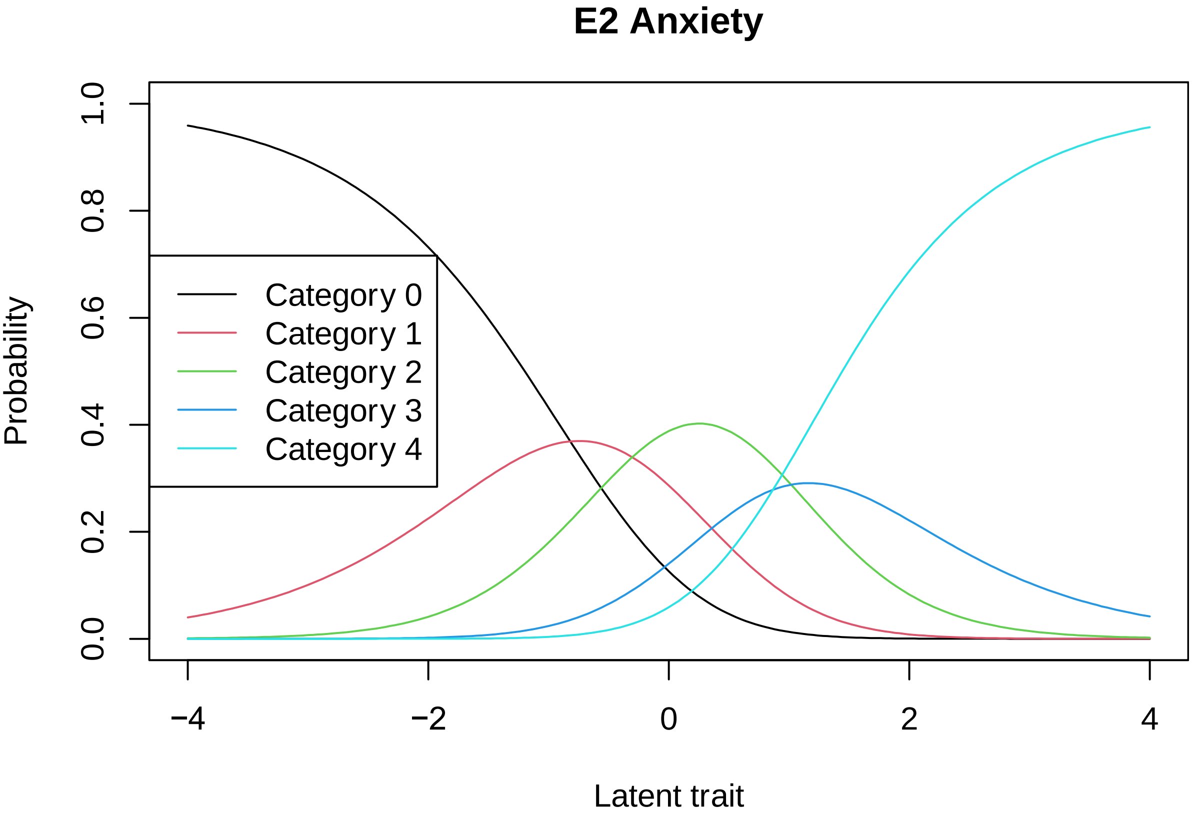
E2 Anxiety
Probability
0.0
0.2
0.4
0.6
0.8
1.0
Category 0
Category 1
Category 2
Category 3
Category 4
−4
−2
0
2
4
Latent trait

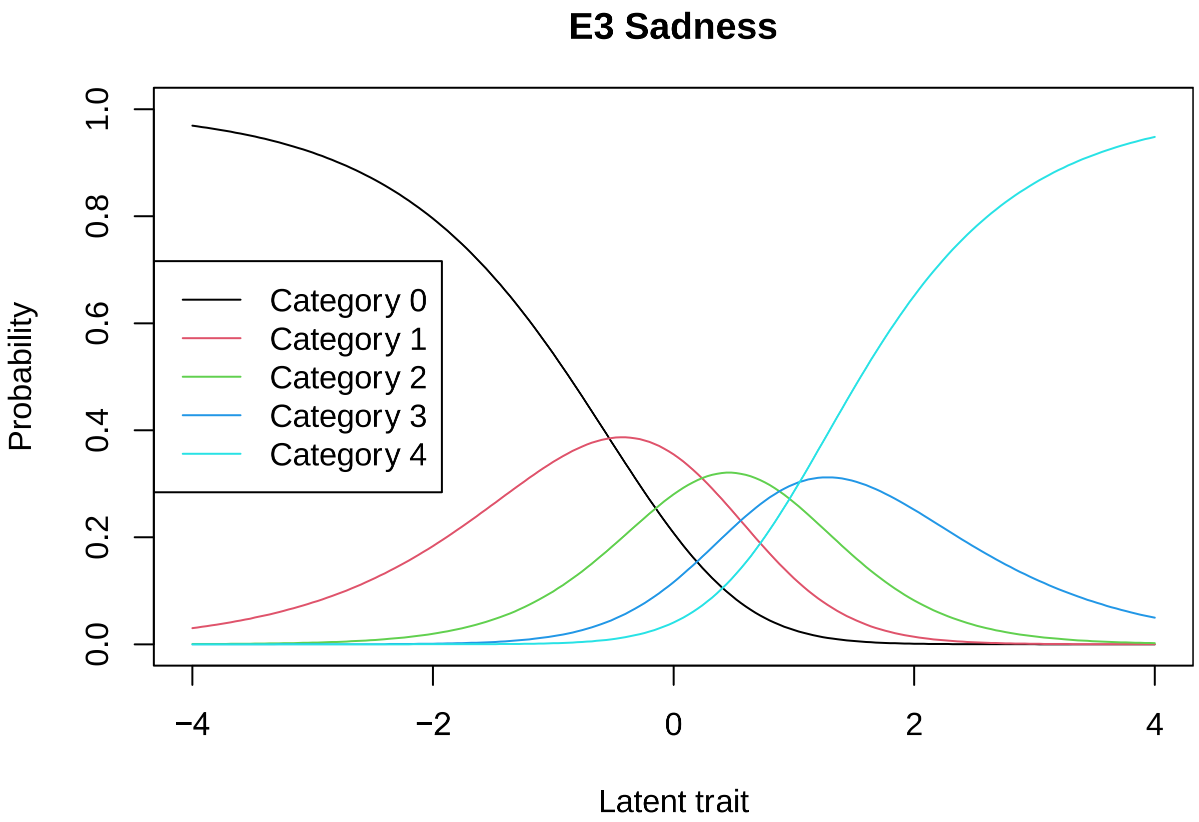
E3 Sadness
Probability
1.0
0.8
0.6
0.4
0.2
0.0
Category 0
Category 1
Category 2
Category 3
Category 4
−4
−2
0
2
4
Latent trait

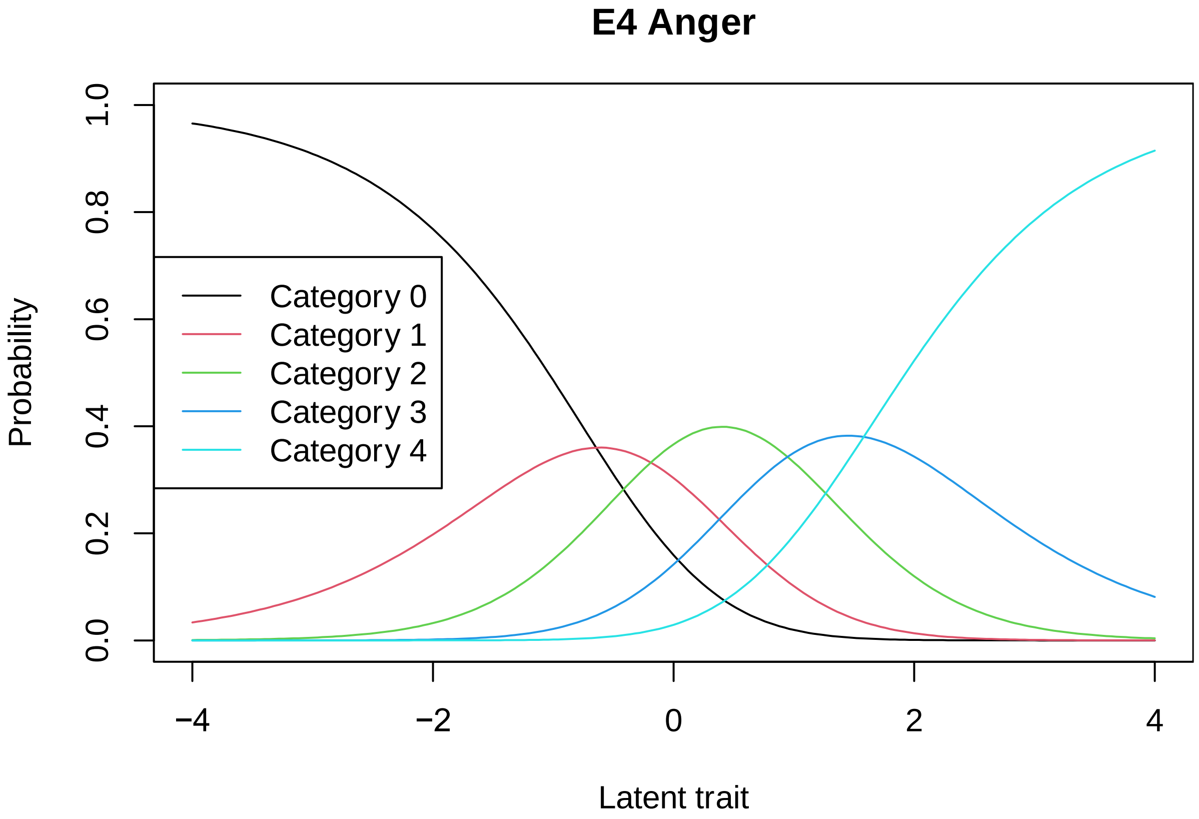
E4 Anger
Probability
1.0
0.8
0.6
0.4
0.2
0.0
Category 0
Category 1
Category 2
Category 3
Category 4
−4
−2
0
2
4
Latent trait

F: Physical

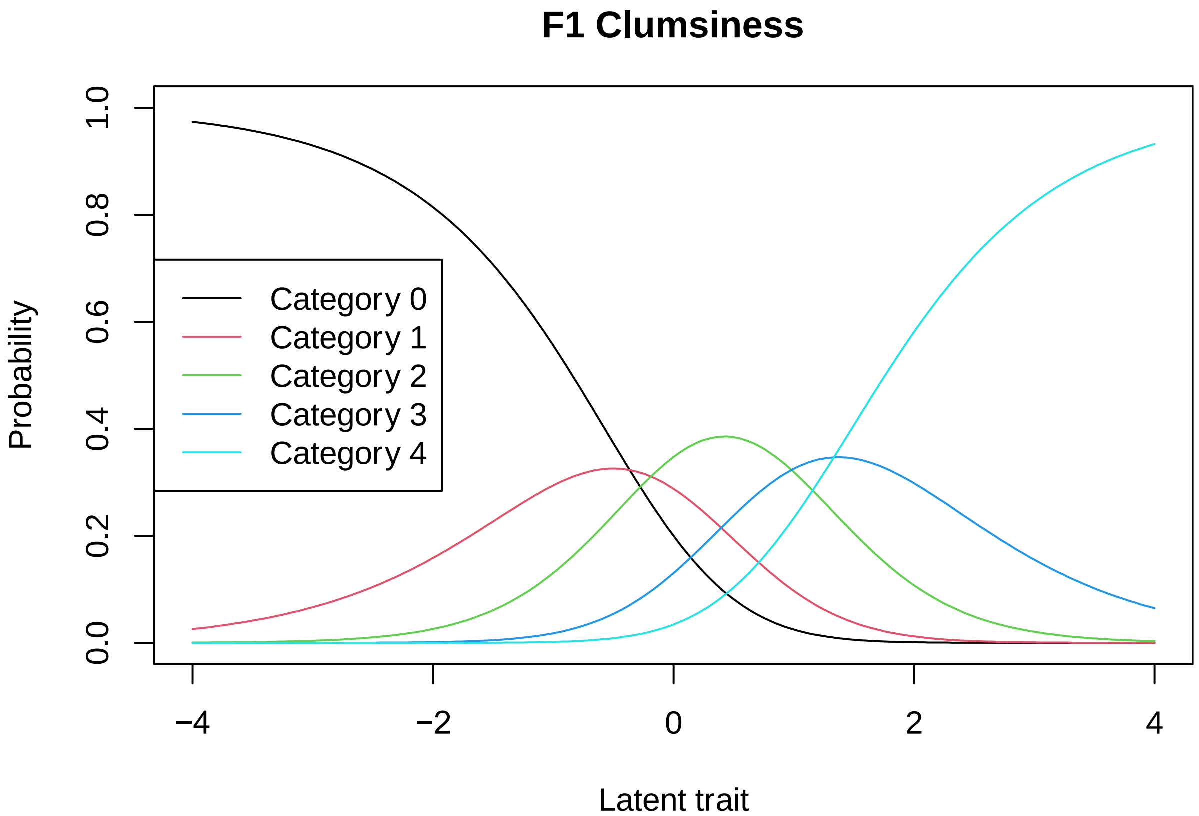
F1 Clumsiness
Probability
0.0
0.2
0.4
0.6
0.8
1.0
−4
−2
0
2
4
Latent trait
Category 0
Category 1
Category 2
Category 3
Category 4

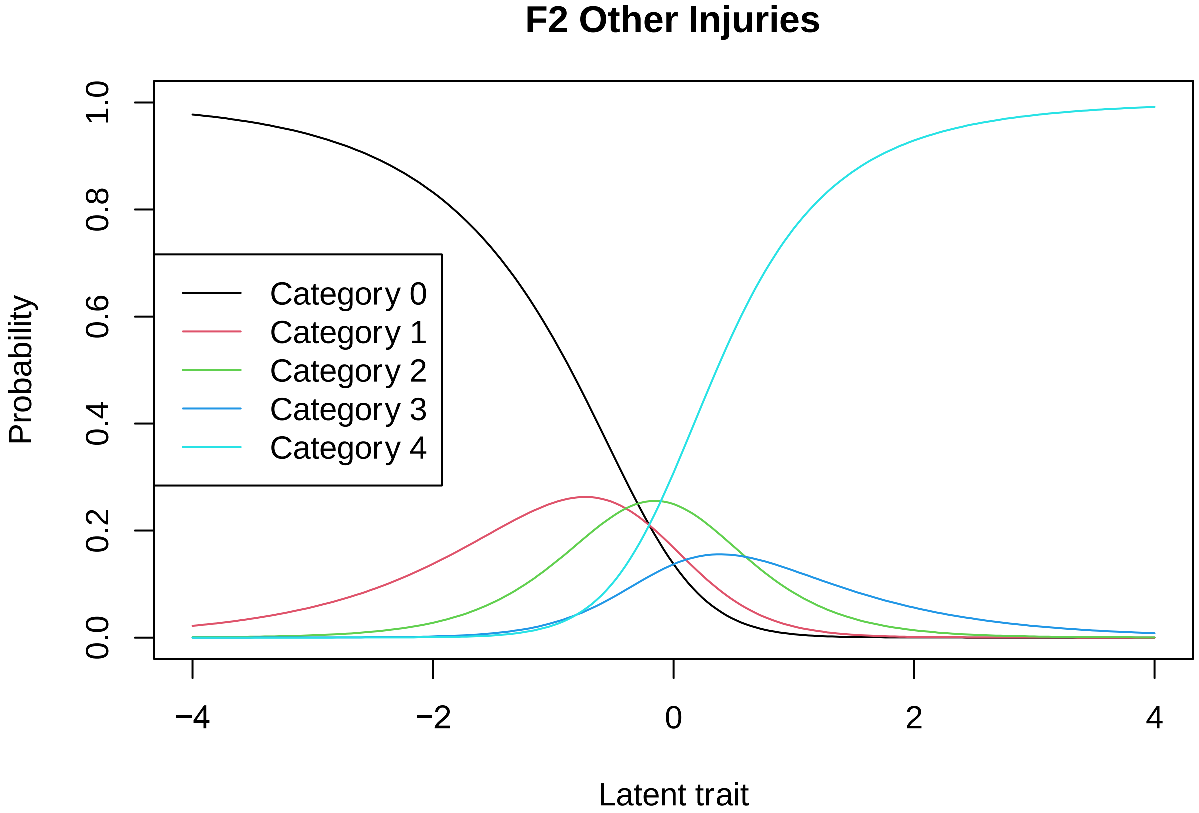
F2 Other Injuries
Probability
0.0
0.2
0.4
0.6
0.8
1.0
−4
−2
0
2
4
Latent trait
Category 0
Category 1
Category 2
Category 3
Category 4

F3 Headaches

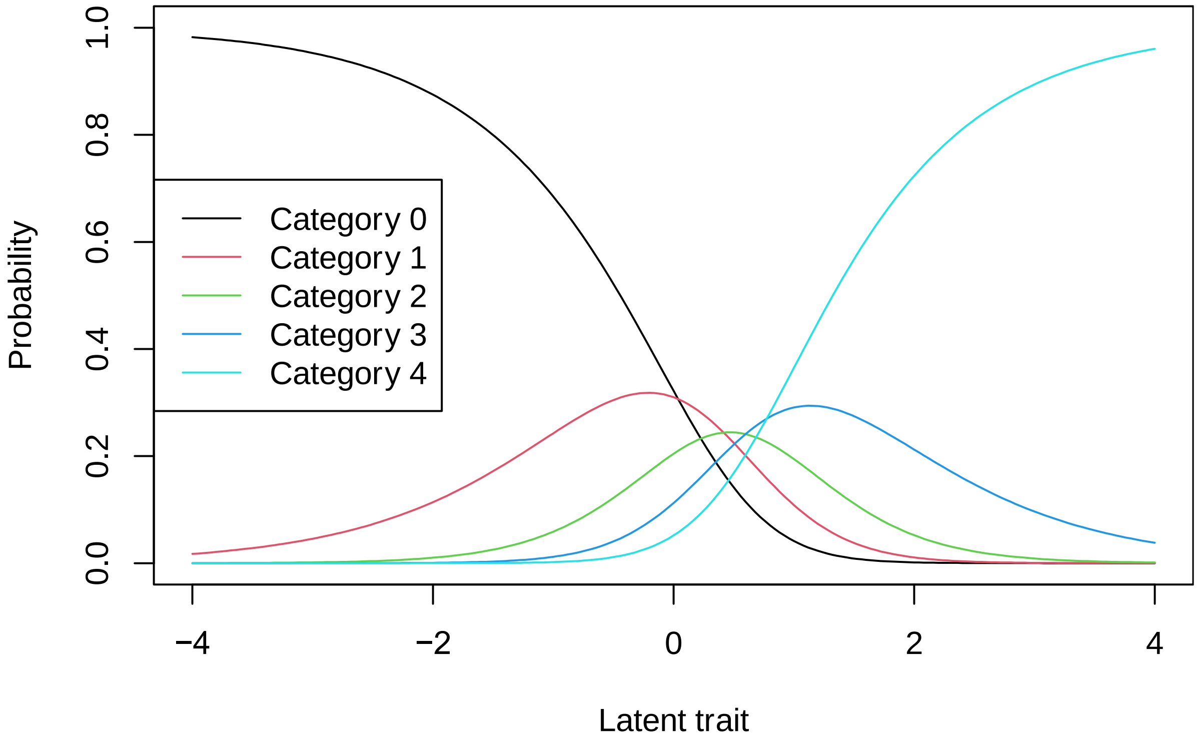

F4 Pain

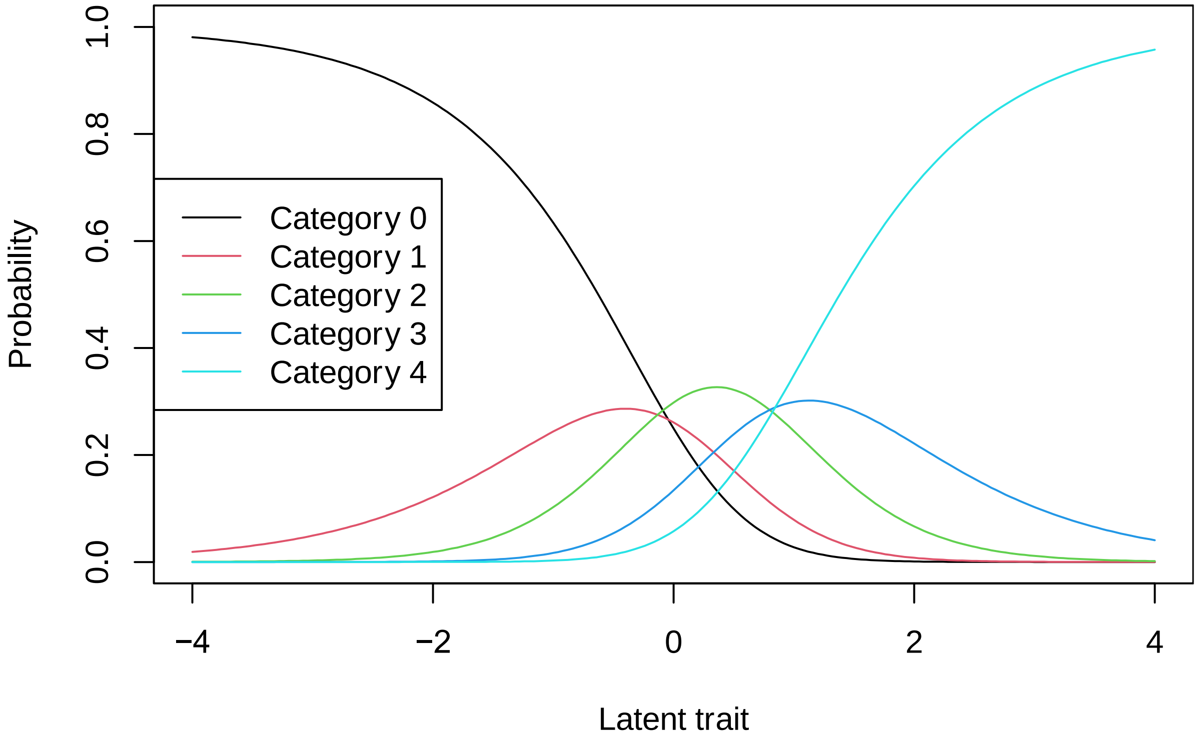

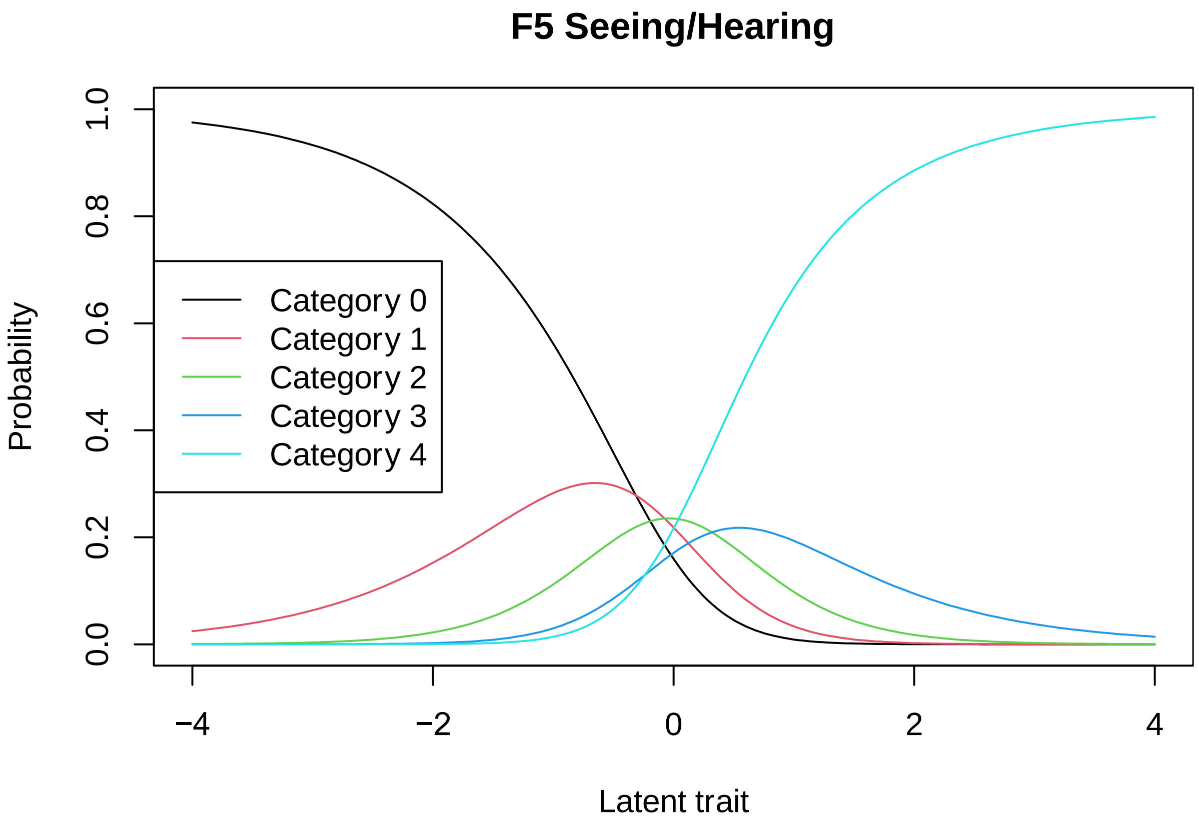
F5 Seeing/Hearing
Probability
Latent trait
Category 0
Category 1
Category 2
Category 3
Category 4
0.0
0.2
0.4
0.6
0.8
1.0
−4
−2
0
2
4

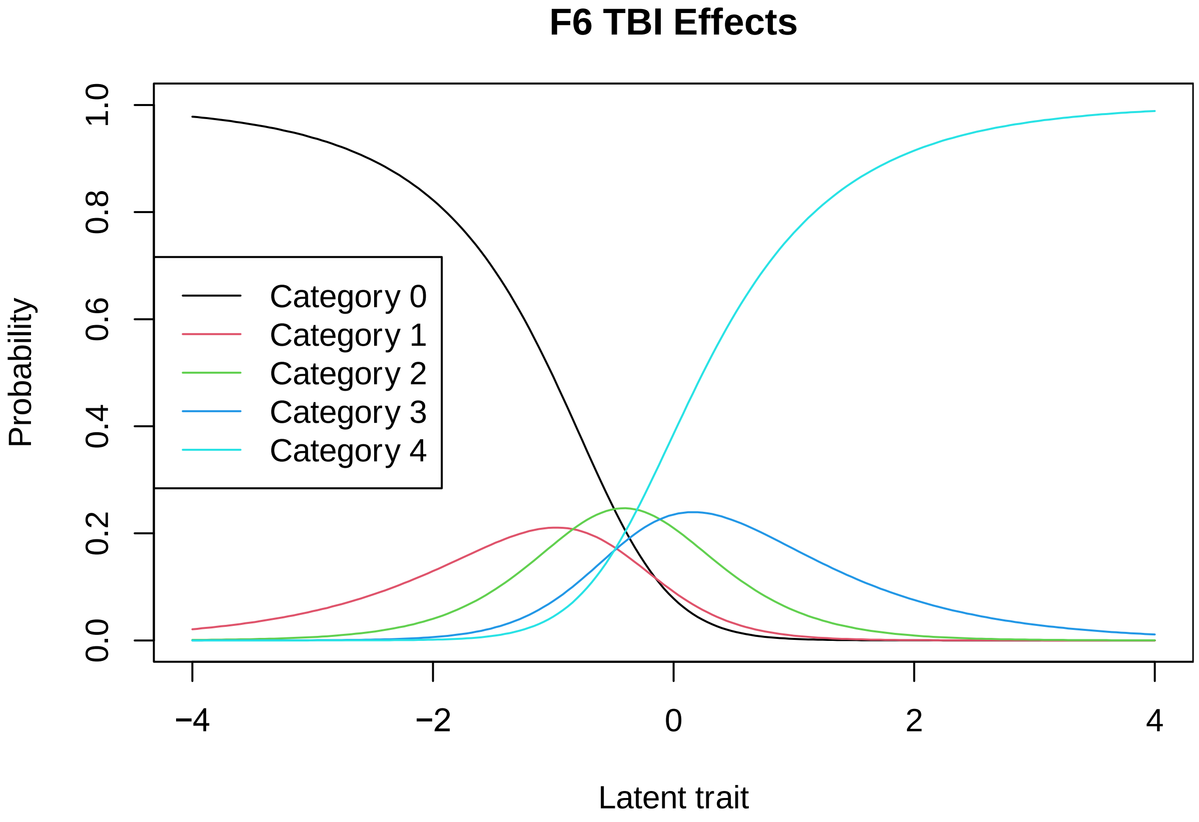
F6 TBI Effects
Probability
Latent trait
Category 0
Category 1
Category 2
Category 3
Category 4
0.0
0.2
0.4
0.6
0.8
1.0
−4
−2
0
2
4
